# Autophagy Differentially Regulates Insulin Production and Insulin Sensitivity

**DOI:** 10.1016/j.celrep.2018.05.032

**Published:** 2018-06-12

**Authors:** Soh Yamamoto, Kenta Kuramoto, Nan Wang, Xiaolei Situ, Medha Priyadarshini, Weiran Zhang, Jose Cordoba-Chacon, Brian T. Layden, Congcong He

**Affiliations:** 1 Department of Cell and Molecular Biology, Feinberg School of Medicine, Northwestern University, Chicago, IL 60611, USA; 2 Department of Microbiology, Sapporo Medical University School of Medicine, Sapporo 060-8556, Japan; 3 Key Laboratory of Industrial Microbiology, Ministry of Education and Tianjin City, College of Biotechnology, Tianjin University of Science and Technology, Tianjin 300457, China; 4 Department of Medicine, Division of Endocrinology, Diabetes and Metabolism, University of Illinois at Chicago, Chicago, IL 60612, USA; 5 Research and Development Division, Jesse Brown Veterans Affairs Medical Center, Chicago, IL 60612, USA

## Abstract

Autophagy, a stress-induced lysosomal degradative pathway, has been assumed to exert similar metabolic effects in different organs. Here, we establish a model where autophagy plays different roles in insulin-producing β cells versus insulin-responsive cells, utilizing knockin (Becn1^F121A^) mice manifesting constitutively active autophagy. With a high-fat-diet challenge, the autophagy-hyperactive mice unexpectedly show impaired glucose tolerance, but improved insulin sensitivity, compared to mice with normal autophagy. Autophagy hyperactivation enhances insulin signaling, via suppressing ER stress in insulin-responsive cells, but decreases insulin secretion by selectively sequestrating and degrading insulin granule vesicles in β cells, a process we term “vesicophagy.” The reduction in insulin storage, insulin secretion, and glucose tolerance is reversed by transient treatment of autophagy inhibitors. Thus, β cells and insulin-responsive tissues require different autophagy levels for optimal function. To improve insulin sensitivity without hampering secretion, acute or intermittent, rather than chronic, activation of autophagy should be considered in diabetic therapy development.

## In Brief

Yamamoto et al. report that, in response to a high-fat-diet challenge, Becn1-mediated autophagy hyperactivation increases insulin sensitivity by reducing ER stress but decreases insulin secretion and storage via autophagic degradation of insulin granules. The results reveal differing roles of autophagy on metabolic regulation in insulin-responsive cells versus insulin-secreting β cells.

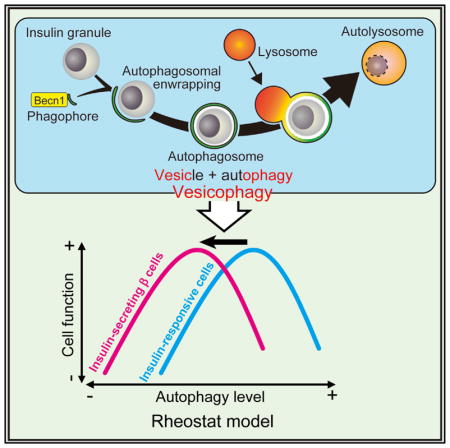

## INTRODUCTION

Diabetes mellitus (DM) is one of the most prevalent diseases worldwide, where the number of individuals with DM is rapidly increasing from 180 million in 1980 to 422 million in 2014 (NCD Risk Factor Collaboration, 2016). In DM, type 2 diabetes (T2D), often accompanied by obesity, represents 85%–95% of the total cases (NCD Risk Factor Collaboration, 2016; Kahn et al., 2006; Prentki and Nolan, 2006). T2D is characterized by hyperglycemia, caused by insulin resistance in the peripheral metabolic tissues and impaired insulin secretion from β cells. However, due to the complexity of the disease, the understanding of the pathogenic mechanisms is still inadequate, and treatment methods are inefficient for many patients (Kahn et al., 2006).

Recently, malfunction of autophagy, an essential stress-induced lysosomal degradation pathway, has been linked to increased incidences of T2D (Ebato et al., 2008; He et al., 2013; Jung et al., 2008; Rocchi and He, 2015). During autophagy, autophagosomal vesicles enwrap unnecessary bulk cytosol, damaged organelles, and/or misfolded proteins and deliver them to lysosomes for breakdown (He and Klionsky, 2009; Mizushima and Komatsu, 2011; Nixon, 2013). Both sedentary lifestyle and overly rich nutrition, the two factors contributing to imbalance of energy intake/expenditure and the global prevalence of obesity and T2D, impair the autophagy activity, whereas fasting and physical exercise, two effective methods to prevent diabetes, can potently induce autophagy (Grumati et al., 2011; He et al., 2012a, 2012b; Liu et al., 2009; Mizushima et al., 2004). Thus, autophagy may mediate the beneficial effects of fasting and exercise against diabetes, and it is intriguing to investigate whether and how stimulation of autophagy may protect against T2D.

Although emerging evidence linked autophagy upregulation with potential metabolic benefits, the therapeutic effect and function of autophagy activation remain enigmatic, and little is known about the tissue-specific requirement of autophagy in metabolic regulation in different types of cells (for example, β cells versus insulin-responsive tissues). In addition, maintenance of healthy glucose metabolism requires coordination of insulin-producing β cells with other insulin-responsive metabolic organs, such as muscle, liver, and adipose tissues. However, it is unclear whether and how autophagy coordinates their functions in the progression of diabetes. Pancreatic β cell dysfunction is a key contributor of various types of diabetes, including type 1 and T2D and maturity-onset diabetes of the young. Basal autophagy has been suggested to be essential for β cell maintenance. β cell-specific knockout of the autophagy gene *Atg7* in mice leads to impaired glucose tolerance and glucose-stimulated insulin secretion (GSIS), morphological abnormalities of islets, and decreased β cell mass (Ebato et al., 2008; Jung et al., 2008; Quan et al., 2012). The decline in islet size is likely caused by accumulation of ubiquitinated proteins and apoptosis induction. However, these findings are based on loss-of-function studies of basal autophagy, and little is known about the physiological consequences of autophagy hyperactivation in β cells or insulin-responsive tissues.

To systematically study the role of autophagy in T2D, we utilized a mouse model that manifests constitutively active autophagy even without treatment of autophagy inducers, caused by a knockin point mutation in the essential autophagy gene *Becn1/Beclin 1* (Rocchi et al., 2017). Unexpectedly, we found that, in response to a high-fat-diet (HFD) challenge, these autophagy-hyperactive mice are more glucose intolerant—but, at the same time, more insulin sensitive—than mice with normal levels of autophagy. We further discovered that chronic hyperactivation of autophagy increases insulin sensitivity in insulin-responsive tissues but reduces insulin storage and secretion by sequestration of insulin granules in β cells by autophagosomes through a process we characterize as “vesicophagy” (Figure 6E). Thus, we propose a model in which autophagy exerts different effects on β cells versus insulin-responsive tissues (Figure 7G). The resulting knowledge will be important for the development of therapies involving modulating autophagy, and we propose that transient or periodic induction of autophagy may be a future treatment of T2D.

## RESULTS

### Becn1^F121A^ Knockin Mice Have Hyperactive Autophagy in Metabolic Tissues, Including Islets

**Figure 1:**
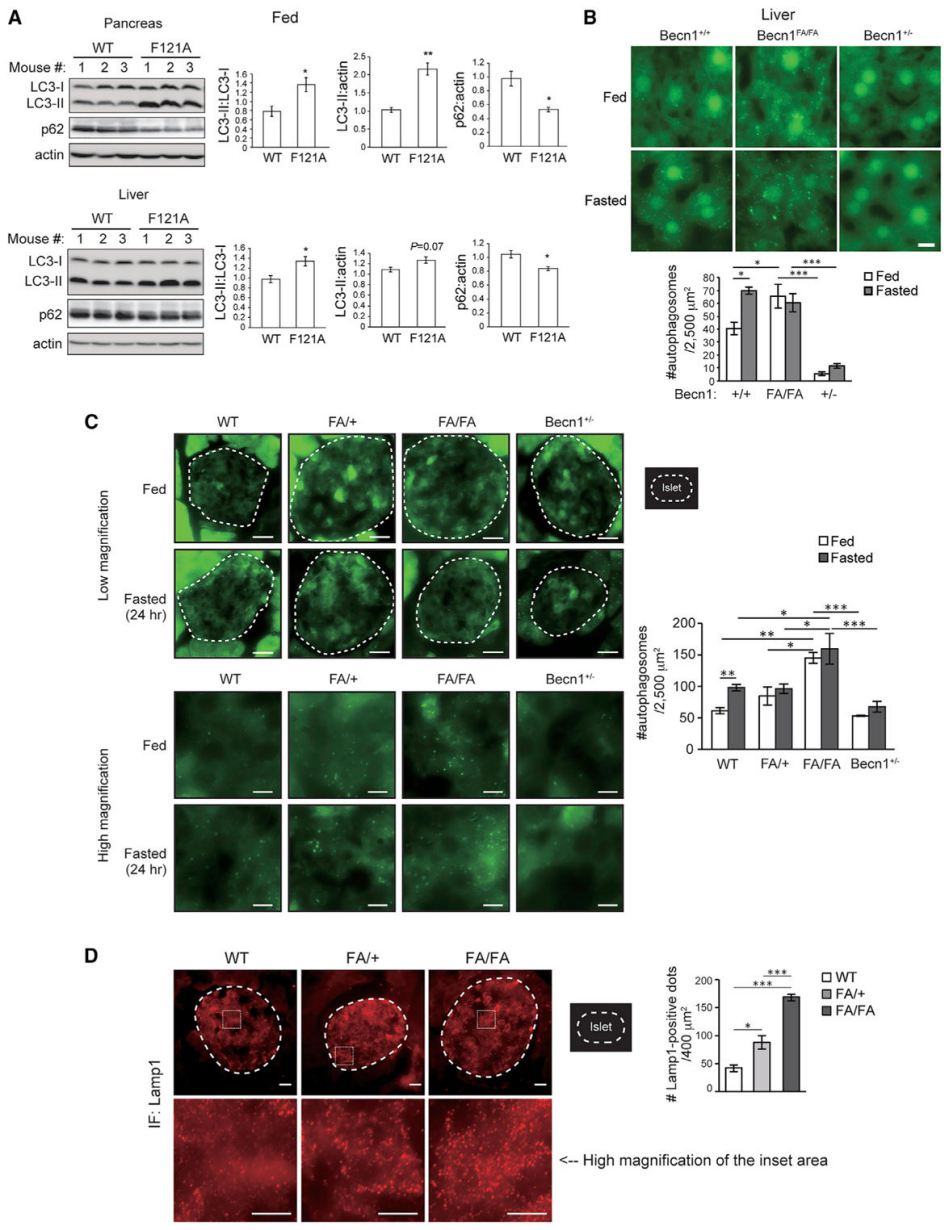
Becn1^F121A^ Knockin Mice Demonstrate Systemic High Autophagy in Metabolic Tissues at Non-autophagy-Inducing Conditions (A) Western blot analysis (left) and quantification (right) of autophagy in pancreas (upper) and liver (lower) of WT and Becn1^F121A^ mice under the fed condition. n = 3; t test. (B) Fluorescence images (upper panels) and quantification (lower panels) of GFP-LC3 puncta (autophagosomes) in the liver of Becn1^+/+^, Becn1^FA/FA^, or Becn1^+/−^ KO mice expressing the GFP-LC3 transgene under fed conditions or subject to 24-hr fasting. FA, F121A. Scale bar, 10 μm. One-way ANOVA. n = 3. (C) Fluorescence images (left: upper panels, low magnification; lower panels, high magnification) and quantification (right) of GFP-LC3 puncta (autophagosomes) in islets of Becn1^+/+^ (WT), Becn1^FA/+^ (FA/+), Becn1^FA/FA^ (FA/FA), or Becn1^+/−^ KO mice expressing the GFP-LC3 transgene under fed conditions or subject to 48-hr starvation. Dashed lines indicate the area of islets, which were detected by anti-insulin staining. FA, Becn1^F121A^. Scale bars, 40 μm in the upper panels and 10 μm in the lower panels. n = 3 mice. The value of each mouse is an average of 8–11 islets per mouse. One-way ANOVA with Tukey-Kramer test. (D) Immunofluorescence images (left) and quantification (right) of Lamp-1 positive lysosomes or autolysosomes in islets of WT, FA/+, and FA/FA mice. Islets were detected by immunostaining with an anti-glucagon antibody. Scale bars, 20 μm in the upper panel and 10 μm in the lower panel, respectively. n = 3–5 mice. The value of each mouse is an average of 5 islets per mouse. One-way ANOVA with Tukey-Kramer test. Results represent mean ± SEM. *p < 0.05; **p < 0.01; ***p < 0.001. See also Figures S1 and S4.

To study how autophagy regulates glucose metabolism, we utilized a knockin mouse line that we recently generated, which contains a point mutation of phenylalanine to alanine (F121A) in the BH3 (Bcl-2 homology 3) domain of the autophagy gene *Becn1/Beclin 1* (Rocchi et al., 2017). This knockin mutation prevents Becn1 from binding with its inhibitor Bcl-2 and leads to constitutively active autophagy in brain and skeletal muscle (Rocchi et al., 2017), which represents a physiological regulatory mechanism of Becn1. Here, we found that, besides brain and muscle, Becn1^F121A^ knockin mice showed increased basal autophagy in liver and islets in the absence of autophagy inducers (Figure 1). Upon autophagy induction, the autophagosome marker protein LC3 is converted from cytosolic non-lipidated form (LC3-I) to autophagosome-associated lipidated form (LC3-II), which can be resolved by western blot or visualized as fluorescent puncta (Mizushima et al., 2004). In addition, degradation of the autophagy cargo receptor p62 serves as another marker of autophagy activation. In homozygous Becn1^F121A^ knockin (Becn1^FA/FA^) mice, we observed conversion of LC3-I to LC3-II and reduction of p62 in liver and pancreas (Figure 1A), suggesting that basal autophagy is induced in these two organs. Upon fasting, Becn1^F121A^ mice also showed further reduction in p62 in both pancreas and liver, compared to wild-type (WT) mice (Figure S1); yet the relative level of LC3-II was reduced in Becn1^F121A^ mice, which is likely through higher autophagy degradation under fasting conditions.

To further investigate autophagy activation in liver and pancreatic islets, we crossed Becn1^F121A^ knockin mice with transgenic mice expressing GFP-tagged LC3. 24-hr fasting significantly increased the number of autophagosomes (represented by GFP-LC3 puncta) in liver and islets of WT mice, and the fasting-induced autophagy was blunted in the autophagy-deficient Becn1^+/−^ knockout (KO) mice (Qu et al., 2003) (Figures 1B and 1C). We found that under normal feeding conditions, there is a trend of elevated autophagy (GFP-LC3 puncta) in the islets of heterozygous Becn1^FA/+^ mice and a dramatically higher level of autophagy in both the liver and islets of the homozygous Becn1^FA/FA^ mice, which is not additionally induced by fasting (Figures 1B and 1C). In addition, both Becn1^FA/+^ and Becn1^FA/FA^ mice showed an increased number of Lamp1-positive autolysosomal structures inside the islets compared to WT mice (Figure 1D), suggesting that the Becn1^F121A^ knockin mutation leads to increased basal autophagy in islets in the absence of autophagy inducers. Overall, Becn1^F121A^ mice showed systemic autophagy hyperactivation in metabolic tissues, including islets.

### Autophagy-Hyperactive Becn1^F121A^ Mice Have Improved Insulin Sensitivity but Impaired Glucose Tolerance in Response to HFD Challenge

**Figure 2:**
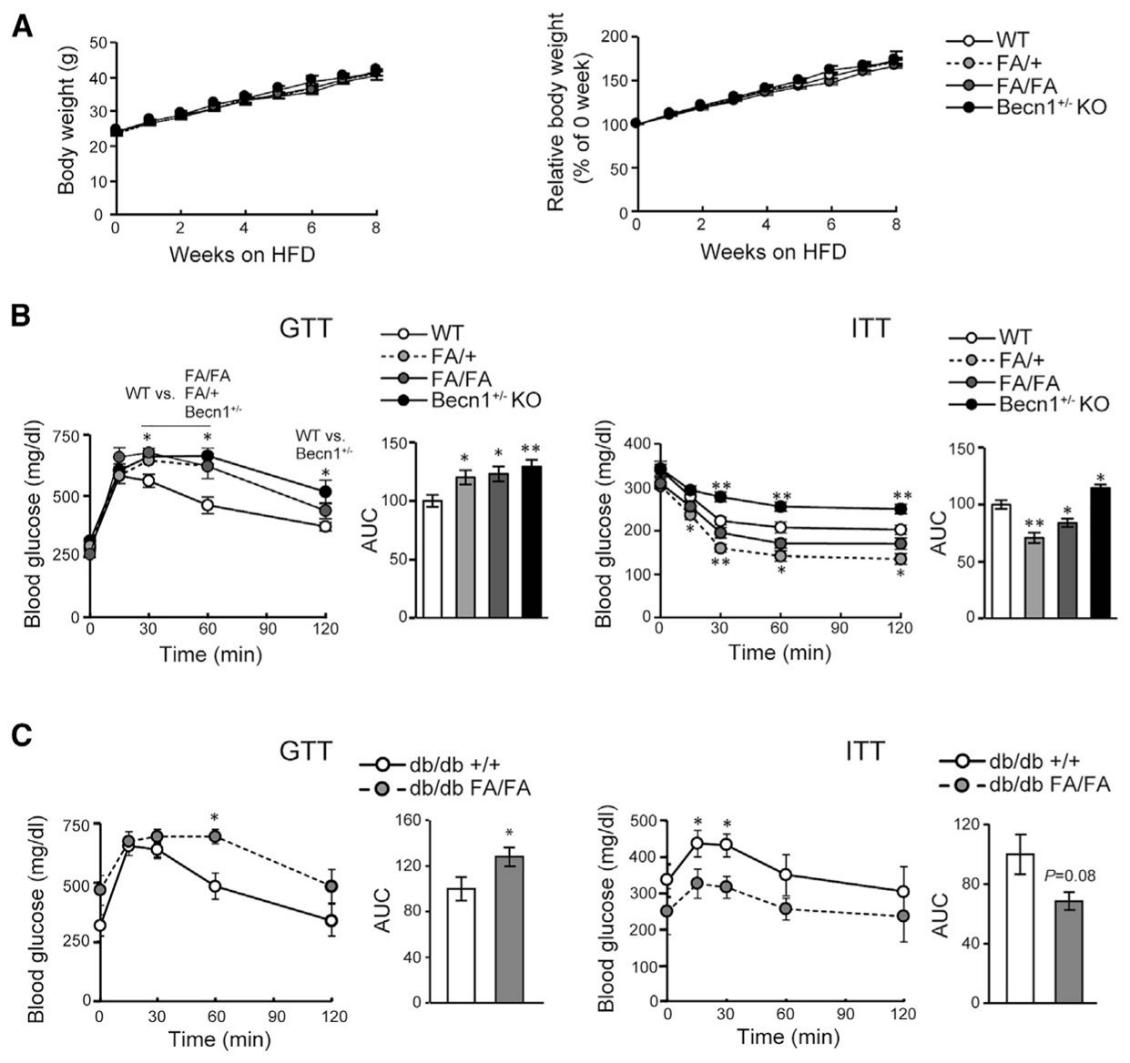
Autophagy-HyperactiveBecn1^F121A^ Mice Are Less Glucose Tolerant, but More Insulin Sensitive, in Response to HFD (A) Body weight gain (in grams in the left panel and percent in the right panel) of Becn1^+/+^ (WT), Becn1^FA/+^ (FA/+), Becn1^FA/FA^ (FA/FA), and Becn1^+/−^ KO mice fed with an HFD for 8 weeks starting at 8 weeks old. WT, n = 34; FA/+, n = 17; FA/FA, n = 22; Becn1^+/−^, n = 15. (B) Glucose tolerance test (GTT, left) and insulin tolerance test (ITT, right) of Becn1^+/+^ (WT), Becn1^FA/+^ (FA/+), Becn1^FA/FA^ (FA/FA), and Becn1 KO mice fed with an HFD for 8 weeks. Statistics are compared with those for WT; one-way ANOVA with Dunnett’s test. n = 6–12. AUC, area under the curve. (C) GTT (left) and ITT (right) in db/db mice expressing WT Becn1 (+/+) or Becn1^F121A^ (FA/FA) treated with HFD. GTT was performed after 4 weeks of HFD feeding, and ITT was performed after 6 weeks of HFD feeding. Statistics are compared with those for db/db +/+ mice. n = 8; t test. Results represent mean ± SEM. *p < 0.05; **p < 0.01. See also Figures S2, S3, and S4.

We next used the Becn1^F121A^ mouse model as a tool to study the physiological consequences of autophagy stimulation in metabolism. When fed a regular diet (RD), both 8-week-old and 16-week-old Becn1^F121A^ mice had a normal fasting glucose level, glucose tolerance, insulin response, and insulinogenic index as compared with WT mice (Figures S2A–S2D). To study whether genetic hyperactivation of autophagy exerts beneficial effects on diabetes, we treated the mice with an HFD for 8 weeks and performed glucose tolerance tests (GTTs) and insulin tolerance tests (ITTs). Under HFD feeding, Becn1^F121A^ mice had similar body weight gain as that of WT mice (Figure 2A). Surprisingly, via GTT, we found that, after HFD feeding, the autophagy-hyperactive Becn1^FA/+^ or Becn1^FA/FA^ mice have exacerbated glucose intolerance compared to WT mice, which is similar to findings with the autophagy-deficient Becn1^+/−^ KO mice (Figure 2B). Interestingly, despite their glucose intolerance in GTT, homozygous Becn1^FA/FA^ or heterozygous Becn1^FA/+^ mice had an elevated insulin response in the ITT compared to WT mice; this phenomenon is different from that of the autophagy-deficient Becn1^+/−^ KO mice, which are not only more glucose intolerant but also more insulin resistant than WT mice (Figure 2B). These data suggest that glucose intolerance observed in the Becn1^F121A^ mice may not be caused by peripheral insulin resistance. To further test this hypothesis, in addition to the HFD-induced T2D model, we examined the effect of Becn1^F121A^-mediated autophagy hyperactivation in db/db mice, a genetic model of obesity and T2D lacking the functional leptin receptor (Chen et al., 1996; King, 2012). We crossed the db/db mice with Becn1^F121A^ mice and performed GTT and ITT experiments on the resulting db/db; Becn1^F121A^ mice. Consistent with HFD-treated mice, db/db; Becn1^F121A^ mice are also less glucose tolerant but more insulin sensitive than db/db; Becn1^WT^ mice (Figure 2C), further supporting our hypothesis that Becn1^F121A^ mice are not insulin resistant.

Consistent with the comparable body weight of HFD-fed WT and Becn1^F121A^ mice, except in liver, we did not detect reduced organ weight or size in Becn1^F121A^ mice, including skeletal muscle, pancreas, brown adipose tissue, and white adipose tissue (Figures S3A and S3B). Although we did not detect significant alterations in glycogen contents, lipid contents (triglycerides and cholesterol) (Figure S3C), lipid droplets (oil red O staining and electron microscopy [EM]) (Figures S3E and S4A), or morphology (H&E staining) (Figure S3F) of the liver from HFD-fed Becn1^F121A^ mice, compared to HFD-fed WT mice, we did observe enhanced sequestration of bulk cytosol by autophagosomes and autolysosomes in the liver from Becn1^F121A^ mice by EM (Figure S4A). In addition, by immunoprecipitating intact autophagosomes, we detected robust selective autophagy of organelles in the liver of Becn1^F121A^ mice, such as mitophagy, revealed by enhanced autophagosomal incorporation of the mitochondrial protein Tom20 (Figure S4B). Thus, we propose that the decline in liver mass in Becn1^FA/+^ and Becn1^FA/FA^ mice may possibly be caused by higher autophagic turnover of organelles and bulk cytosol. Furthermore, to determine whether autophagy hyperactivation interferes with hepatic function and causes possible hepatic injury, we measured the level of triglycerides, cholesterol, NEFAs (non-esterified fatty acids), albumin, and ALT (alanine transaminase) in the serum of HFD-fed WT and Becn1^F121A^ mice. We found that there is no significant difference in the level of the aforementioned circulating parameters (Figure S3D), suggesting that the autophagy-hyperactive Becn1^F121A^ mice do not have apparent hepatic impairment, compared to WT mice. Thus, altogether, these data suggest that Becn1^F121A^-mediated autophagy is still within the physiological range and does not lead to organ atrophy or hepatic impairment.

### Becn1^F121A^ Expression Leads to Reduced ER Stress and Enhanced Insulin Signaling in Insulin-Responsive Tissues

**Figure 3:**
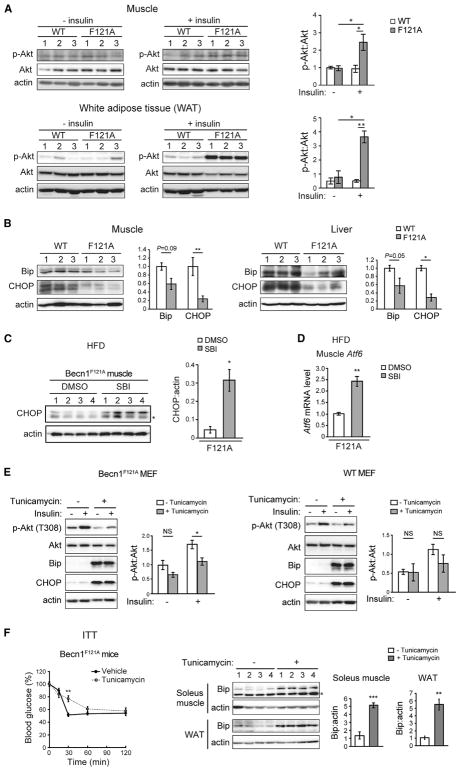
Reduction in HFD-Induced ER Stress in Becn1^F121A^ Mice Mediates Higher Insulin Sensitivity in Insulin-Responsive Tissues (A) Western blot analyses (left) and quantification (right) of insulin-induced Akt T308 phosphorylation in muscle (upper panels) and white adipose tissue (lower panels) of WT and Becn1^F121A^ mice fed with an 8-week HFD. The tissues were collected 10 min after injection i.p. of 2 U/kg insulin. n = 3; one-way ANOVA with Tukey-Kramer test. (B) Reduced HFD-induced ER stress in insulin-responsive tissues of Becn1^F121A^ mice. Western blot analyses and quantification of the ER stress markers Bip and CHOP in skeletal muscle (left) and liver (right) of WT or Becn1^F121A^ mice treated with HFD for 8 weeks. n = 3; t test. (C and D) Western blot analysis and quantification of CHOP (C) and qPCR analysis of *Atf6* (D) in skeletal muscle of Becn1^F121A^ mice treated with HFD for 8 weeks and subject to 10 injections of the autophagy inhibitor SBI-0206965 (SBI) or vehicle (DMSO) once daily during the last 2 weeks of HFD feeding. The asterisk indicates a nonspecific band. n = 4; t test. (E) Western blot analysis and quantification of insulin-stimulated phosphorylation of Akt T308 in WT (right) or Becn1^F121A^ (left) primary MEFs treated with vehicle or tunicamycin at 0.01 μg/ml for 16 h. Samples were collected 10 min after insulin treatment. N = 4. t test. (F) ITT of 8-week HFD-fed Becn1^F121A^ mice subject to one injection of vehicle or the ER stressor tunicamycin at 1 μg/kg for 24 hr. Percentage of blood glucose at the basal level (time 0) during ITT is indicated. n = 5. Western blot analysis and quantification of the ER stress marker Bip in skeletal muscle (soleus) and white adipose tissue (WAT) from the mice are indicated on the left; t test. Results represent mean ± SEM. *p < 0.05; **p < 0.01; ***p < 0.001; NS, not significant. See also Figures S5, S6, and S7.

Besides improved performance in ITT, we examined other markers for insulin sensitivity in insulin-responsive tissues of Becn1^F121A^ mice, including insulin-induced Akt phosphorylation and endoplasmic reticulum (ER) stress. Insulin stimulates phosphorylation of Akt at T308 downstream of the insulin receptor. With regular diet feeding, Becn1^F121A^ mice and WT mice showed comparable Akt phosphorylation after insulin stimulation in skeletal muscle (gastrocnemius) and white adipose tissue (Figures S5A and S5B). We found that HFD treatment inhibited insulin-stimulated Akt phosphorylation in insulin-responsive tissues; however, this inhibition is reversed in both skeletal muscle and white adipose tissue of HFD-fed Becn1^F121A^ mice (Figure 3A). Along this line, after insulin stimulation, we also observed higher phosphorylation of Akt at T308, as well as a trend of higher phosphorylation at S473, in primary MEFs (mouse embryonic fibroblasts) isolated from both Becn1^FA/+^ and Becn1^FA/FA^ mice (Figure S5C). These data suggest that autophagy-hyperactive mice have improved insulin sensitivity in insulin-targeting tissues.

We then analyzed the possible causes of improved insulin sensitivity in HFD-fed Becn1^F121A^ mice. We did not detect significant differences in markers of the inflammasome signaling pathway, including NLRP3, cleaved caspase-1, and cleaved IL-1β, in insulin-responsive tissues (liver and white adipose tissue) of WT and Becn1^F121A^ mice after an HFD challenge (Figure S7A), suggesting that Becn1^F121A^-mediated autophagy activation does not lead to changes in inflammasome activation in insulin-responsive tissues after HFD feeding. However, we found that HFD-induced ER stress is significantly reduced in the insulin-responsive tissues in Becn1^F121A^ mice, as evidenced by suppressed ER chaperones and ER stress markers Bip and CHOP (C/EBP homologous protein) in the skeletal muscle and liver of HFD-fed Becn1^F121A^ mice, compared to HFD-fed WT mice (Figure 3B). Treatment of the autophagy inhibitor SBI-0206965 in HFD-fed Becn1^F121A^ mice restores the levels of the ER stress marker CHOP (Figure 3C) and the ER-stress-induced transcription factor ATF6 (Figure 3D) in skeletal muscle, suggesting that autophagy hyperactivation mediated by Becn1^F121A^ is responsible for the reduction of the ER stress levels in insulin-responsive tissues. These data suggest that Becn1^F121A^-mediated autophagy hyperactivation may enhance insulin signaling through reducing ER stress in insulin-responsive cells, a key factor that causes insulin resistance (Flamment et al., 2012; Hummasti and Hotamisligil, 2010; Salvadó et al., 2015; Zhang et al., 2013). To test this hypothesis, we treated primary MEFs from WT and Becn1^F121A^ mice with an ER stressor, tunicamycin, and analyzed insulin-induced Akt phosphorylation. While, in WT cells, the suppressive effect of tunicamycin on insulin signaling is not significant, in Becn1^F121A^ MEFs, tunicamycin treatment fully restores ER stress and abolishes Becn1^F121A^-mediated insulin sensitization (Figure 3E). In addition, to test the hypothesis *in vivo*, we treated HFD-fed Becn1^F121A^ mice with tunicamycin for 24 hr. As previously reported (Feng et al., 2017), treatment with the ER stressor tunicamycin significantly inhibited gluconeogenesis in liver and reduced the steady-state blood glucose level (Figure S6). Nonetheless, we found that tunicamycin elevates ER stress in both skeletal muscle and white adipose tissue and reverses the improved insulin sensitivity in HFD-fed Becn1^F121A^ mice, represented by the percentage of blood glucose compared to baseline (time 0) in ITTs (Figure 3F). These findings suggest that Becn1^F121A^-mediated reduction in ER stress in insulin-responsive tissues contributes to improved insulin sensitivity in the Becn1^F121A^ knockin mice. Taken together, we concluded that glucose intolerance in Becn1^F121A^ mice is not caused by insulin resistance, since Becn1^F121A^-mediated autophagy hyperactivation improves, rather than impairs, insulin sensitivity in insulin-responsive tissues.

### Becn1^F121A^ Reduces GSIS and Insulin Storage in β Cells

**Figure 4:**
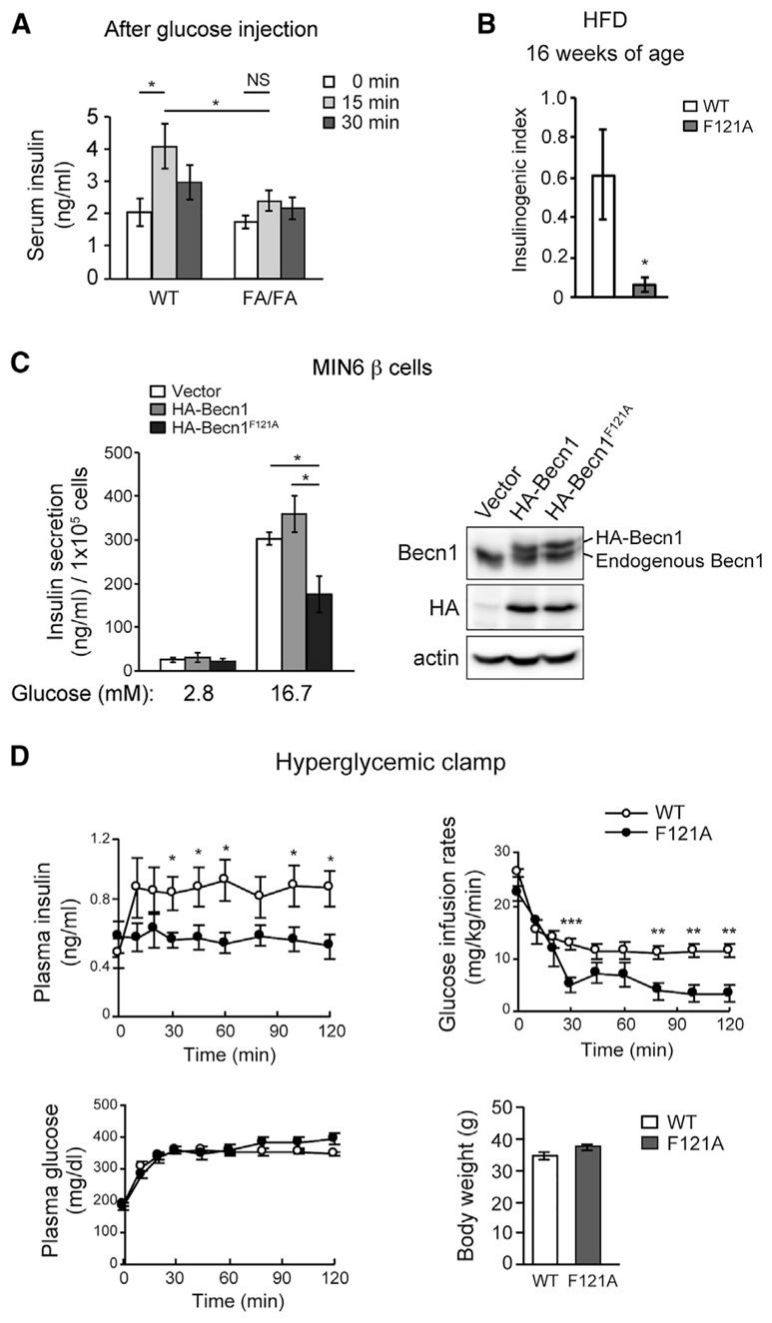
The Autophagy-Hyperactive Becn1^F121A^ Mutation Reduces GSIS (A) ELISA analyses of plasma insulin levels 0–30 min after glucose injection in HFD-fed WT and Becn1^F121A^ mice. n = 6–7; one-way ANOVA with Tukey-Kramer test. (B) Insulinogenic index of WT and Becn1^F121A^ mice fed with HFD for 8 weeks. n = 6. (C) GSIS in MIN6 β cells stably expressing vector, or hemagglutinin (HA)-tagged WT Becn1 or Becn1^F121A^ mediated by lentivirus, normalized to cell number. Western blot analysis of Becn1 expression in MIN6 cells was shown on the right. n = 5; t test. (D) Hyperglycemic clamp study of WT and Becn1^F121A^ mice fed with HFD for 9 weeks. Plasma insulin, glucose infusion rate, plasma glucose, and body weight are shown. n = 9–10; t test. Data represent mean ± SEM. *p < 0.05; **p < 0.01; ***p < 0.001. See also Figure S7.

From the aforementioned data, we reasoned that insulin secretion and/or storage, rather than insulin sensitivity, is impaired in Becn1^F121A^ mice. To test this hypothesis, we first examined GSIS during GTT. Fifteen minutes after glucose injection, the insulin level in the serum was elevated in HFD-fed WT mice; however, plasma insulin fails to elevate in HFD-fed Becn1^F121A^ mice after glucose injection (Figure 4A), which is also reflected by a reduced insulinogenic index in Becn1^F121A^ mice (Figure 4B). In addition, we found that overexpression of Becn1^F121A^ in the MIN6 β cell line reduced GSIS upon high-glucose (16.7-mM) treatment (Figure 4C), suggesting that the Becn1^F121A^ mutation has a dominant-negative effect on GSIS. To directly measure insulin secretion *in vivo*, we performed hyperglycemic clamp studies in HFD-fed WT and Becn1^F121A^ mice. We found that Becn1^F121A^ mice show reduced GSIS into the circulation, leading to deceased glucose infusion *in vivo* (Figure 4D). In contrast to insulin levels, the release of glucagon from pancreatic α cells is not affected in HFD-fed Becn1^F121A^ mice (Figure S7B). These data suggest that Becn1^F121A^-mediated high autophagy reduces insulin secretion from β cells after HFD challenge.

**Figure 5:**
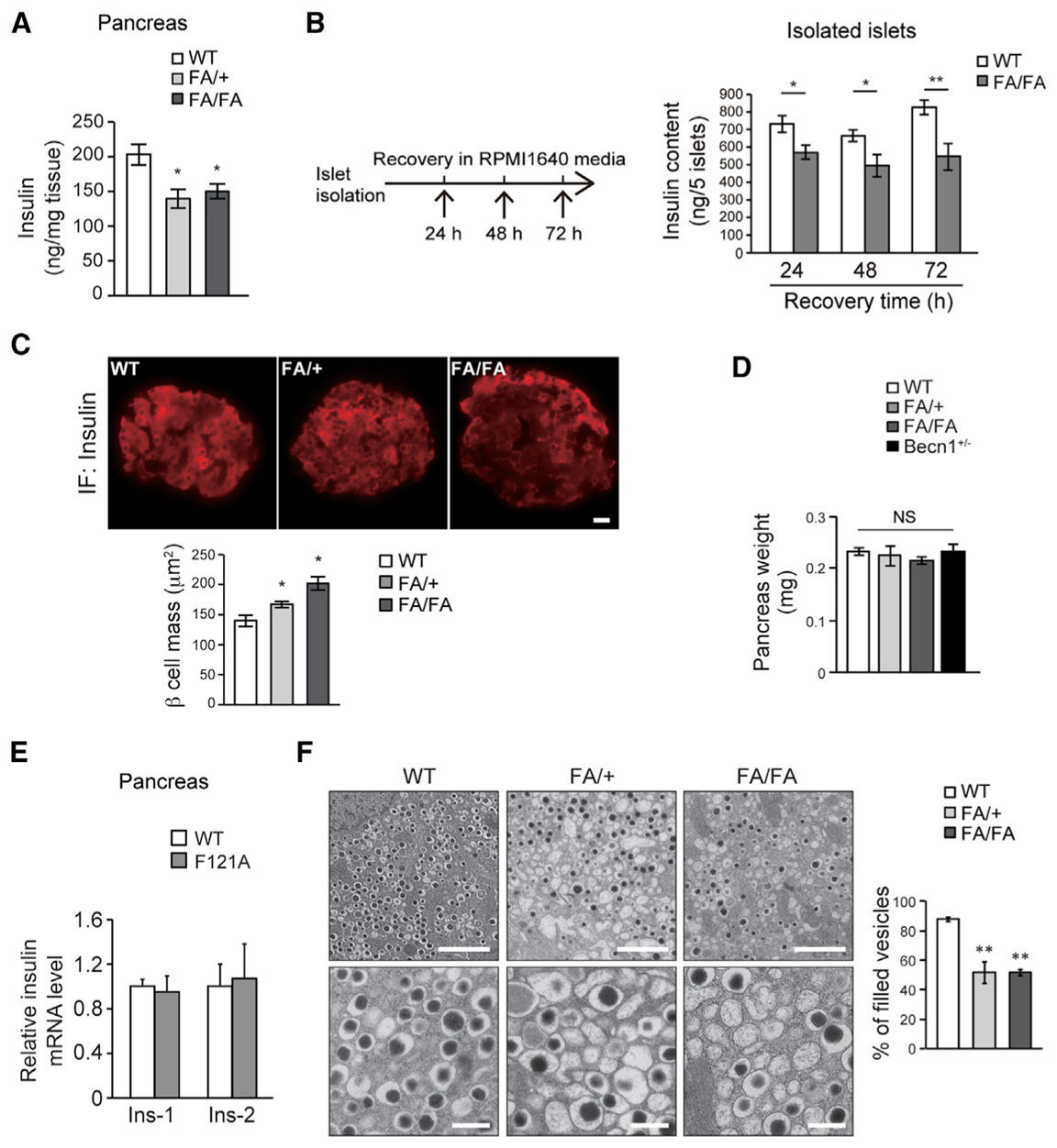
Autophagy-HyperactiveBecn1^F121A^ Mice Have Reduced Pancreatic Insulin Storage in Response to HFD, Despite No Reduction in β Cell or Pancreas Mass (A) ELISA analyses of insulin levels in pancreas of HFD-fed WT, FA/+, and FA/FA mice. n = 3–6; one-way ANOVA with Tukey-Kramer test. (B) ELISA analyses of insulin contents in 5 islets isolated from HFD-fed WT and Becn1^F121A^ mice, in a time course of 24- to 72-hr recovery in RPMI 1640 media after islet isolation. n = 6–10; t test. (C and D) Immunofluorescence images (top) and quantification (bottom) of β cell mass (insulin-positive area) (C) and quantification of whole pancreas weight (D) in Becn1^+/+^ (WT), Becn1^FA/+^ (FA/+), and Becn1^FA/FA^ (FA/FA) mice using anti-insulin antibody. Scale bar, 20 μm. *p < 0.05; NS, not significant; compared with WT. n = 4–6. (E) Real-time PCR analyses of both insulin genes, *Ins-1* and *Ins-2*, in islets of WT or Becn1^F121A^ mice reveal that insulin mRNA transcription is not affected by autophagy activation. n = 3–6. (F) EM images (left) and quantification (right) of insulin granules in islets of WT, FA/+, and FA/FA mice fed with an HFD for 8 weeks. Scale bars, 2 μm in the upper panels and 500 nm in the lower panels. Statistics are compared with WT. n = 3 mice. The value of each mouse is an average of 5 islet areas per mouse; one-way ANOVA with Dunnett’s test. Data represent mean ± SEM. *p < 0.05; **p < 0.01. See also Figure S7.

We then found that decreased insulin secretion in autophagy-hyperactive mice is caused by reduction in pancreatic insulin storage. Under regular diet feeding, Becn1^F121A^ mice showed normal insulin contents in the pancreas by ELISA analyses (Figure S7C). However, after HFD treatment, there was less insulin content in the pancreas of both heterozygous and homozygous Becn1^F121A^ mice (Figure 5A). Furthermore, islets isolated from HFD-fed Becn1^F121A^ mice showed consistently lower insulin contents than those isolated from HFD-fed WT mice, in a time course of 24-, 48-, and 72-hr *ex vivo* culture after isolation (Figure 5B). Notably, the percentage of secreted insulin versus insulin storage in Becn1^F121A^ islets is similar to that of WT islets (Figure S7D), suggesting that the secretory pathway, per se, in Becn1^F121A^ islets is unlikely affected.

Importantly, the reduction in GSIS and insulin contents in autophagy-hyperactive mice is not caused by islet disruption or degeneration, because we found that the pancreatic insulin-positive area (representing β cell mass) is not reduced in Becn1^F121A^ mice in response to an HFD. Instead, there is a 40% increase in insulin-positive islet area in Becn1^F121A^ homozygotes and a 20% increase in heterozygotes, compared to WT mice (Figure 5C), while the weight of whole pancreas remains comparable among all the three genotypes plus Becn1^+/−^ KO mice (Figure 5D), suggesting that autophagy hyperactivation does not reduce β cell mass; rather, it may lead to a compensatory increase.

We then found that the reduction in insulin contents in Becn1^F121A^ mice is not caused by decreased insulin expression either; rather, it is due to increased insulin turnover. The mRNA transcriptional levels of both insulin genes, *Ins1* and *Ins2*, were comparable between WT and Becn1^F121A^ mice (Figure 5E). From ultrastructural analysis on β cells from HFD-fed WT and Becn1^F121A^ mice by EM, we discovered that, in both Becn1^FA/+^ and Becn1^FA/FA^ islets, the number of vesicles packed with high-density mature insulin granules (defined as “filled vesicles”) was dramatically decreased (Figure 5F). Altogether, these data demonstrate that Becn1^F121A^ knockin mice have reduced insulin secretion and insulin storage due to fewer insulin granules in β cells, despite normal insulin mRNA transcription.

### Insulin Granules Are Sequestered and Degraded by Autophagosomes in Autophagy-Hyperactive Mice

**Figure 6:**
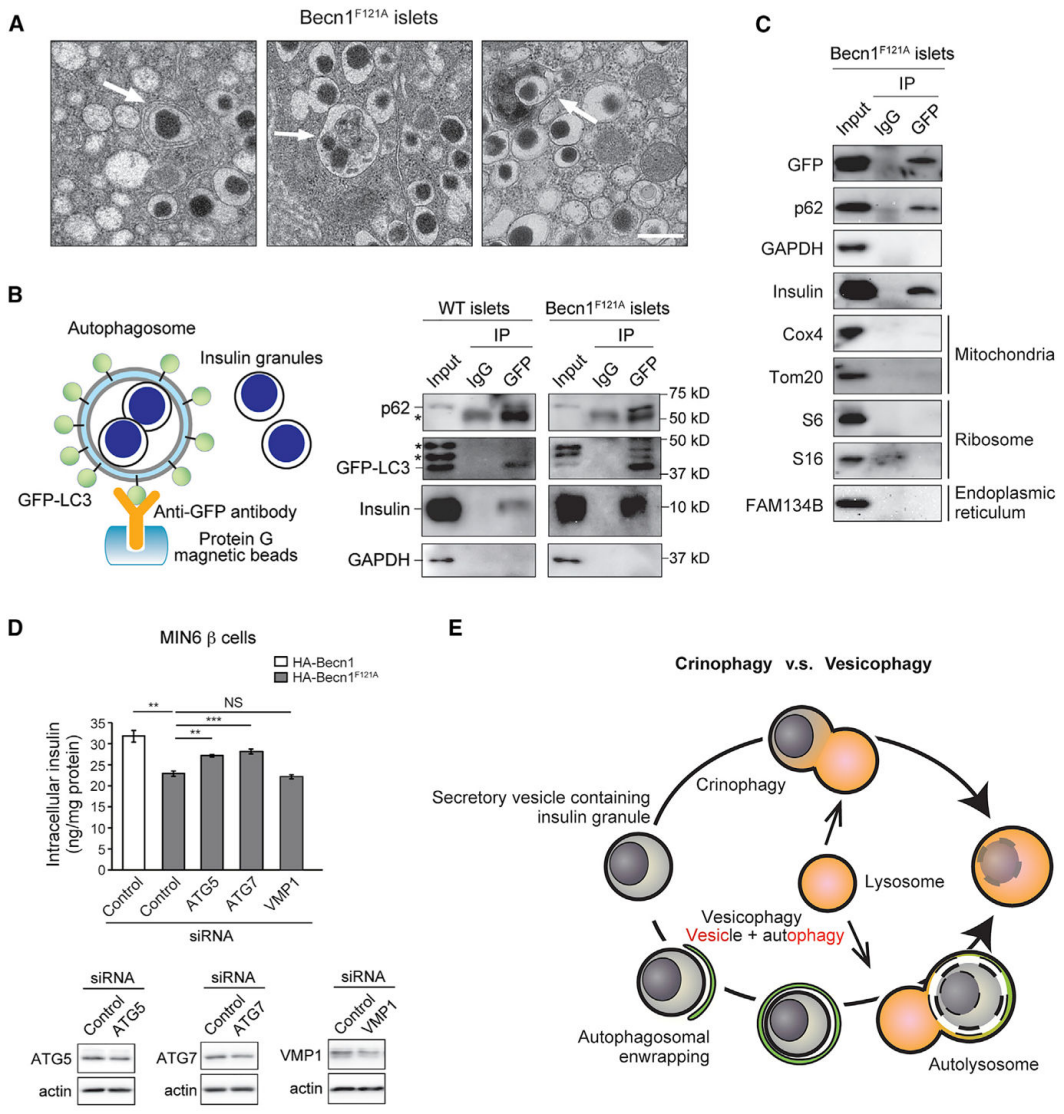
Selective Autophagosomal Sequestration of Insulin Granules in the Islets of Autophagy-Hyperactive Becn1^F121A^ Mice (A) EM analysis of insulin granules enwrapped and degraded in autophagosomes or autolysosomes (indicated by arrows) in islets of Becn1^F121A^ mice. Scale bar, 500 nm. (B) Autophagosomal sequestration of insulin granules is induced in islets of Becn1^F121A^ mice. (Left) Scheme of autophagosome immunoprecipitation by anti-GFP antibody from isolated islets of mice expressing the autophagosome marker GFP-LC3. (Right) Western blot detection of insulin inside isolated autophagosomes from WT or Becn1^F121A^ islets. A known autophagy cargo p62 serves as a positive control. Asterisks indicate nonspecific bands. (C) Western blot detection of cargos of multiple known selective autophagy pathways, including mitophagy (Tom20 and Cox4), ERphagy (Fam134b), and ribophagy (ribosome proteins S6 and S16), in isolated islets from GFP-LC3 Becn1^F121A^ mice. (D) ELISA analyses of insulin contents in MIN6 cells stably expressing Becn1^WT^ or Becn1^F121A^ transfected with control siRNA or siRNA against *Atg5*, *Atg7*, or *Vmp1*. siRNA knockdown efficiency of *Atg5*, *Atg7*, and *Vmp1* in MIN6 β cells was shown below. n = 4; t test. Data represent mean ± SEM. **p < 0.01; ***p < 0.001; NS, not significant. (E) Schematic representation of crinophagy versus vesicophagy. Crinophagy refers to the direct fusion of hormone-containing vesicles with lysosomes, whereas vesicophagy is the autophagosomal sequestration of secretory vesicles containing any secreted granules (not limited to hormones) and the subsequent delivery to lysosomes for degradation.

Thus, we hypothesize that chronic autophagy hyperactivation promotes the degradation of insulin granules in β cells, which leads to impaired glucose tolerance in response to an HFD, as well as compensatory production of immature secretory vesicles. We tested the hypothesis by EM and direct biochemical methods. Through EM, we found that in islets of Becn1^F121A^ mice, insulin granules were frequently present in double-membrane autophagosomes or partially degraded in autolysosomes (Figure 6A), suggesting that insulin granules are efficient cargos of autophagy.

To directly address whether insulin granules are sequestered inside autophagosomes upon autophagy hyperactivation, we immunoisolated intact autophagosomes from isolated islets of WT or Becn1^F121A^ mice expressing the autophagosome marker GFP-LC3, using an anti-GFP antibody and magnetic beads following centrifugation (Gao et al., 2010; Rocchi et al., 2017) (Figure 6B). The purity of autophagosomes is validated by co-isolation of the known autophagy cargo p62 but not a cytosolic enzyme GAPDH (Figure 6B). Using this method, we detected that insulin is co-precipitated with autophagosomes from isolated islets in Becn1^F121A^ mice, and notably, that there is a significantly higher level of insulin immunoprecipitated in autophagy-hyperactive Becn1^F121A^ mice than in WT mice (Figure 6B). These data support our hypothesis biochemically that insulin granules are cargos of autophagosomes and degraded by high autophagy in β cells.

We then found that the autophagic sequestration of insulin-containing vesicles in β cells is a selective process. In autophagosomes immunopurified from isolated islets of Becn1^F121A^ mice, except insulin, we did not detect markers of other known forms of selective autophagy, such as mitophagy (Tom20 and Cox4), ERphagy (Fam134b), and ribophagy (S6 and S16 ribosomal proteins) (Figure 6C). Thus, the autophagic degradation of insulin granules in β cells is different from mitophagy, ERphagy, or ribophagy.

We termed this process as “vesicophagy” (Figure 6E), which describes the autophagic degradation of secretory vesicles that contain various granular contents. Our findings also suggest that vesicophagy is distinct from crinophagy, which is the degradation of insulin granules via direct fusion with lysosomes, independent of macroautophagy (Figure 6E) (Klionsky et al., 2016). We found, instead, that small interfering RNA (siRNA) knockdown of either of the 2 essential autophagy genes, *Atg5* and *Atg7*, partially restores the intracellular insulin storage in MIN6 β cells stably expressing Becn1^F121A^ (Figure 6D). These data suggest that, in contrast to crinophagy, vesicophagy is dependent upon the general autophagy machinery. In addition, we found that, mechanistically, vesicophagy is not similar to zymophagy (Grasso et al., 2011), which refers to the autophagic degradation of zymogen granules in pancreatic acinar cells. The insulin levels in Becn1^F121A^ cells are not restored by knockdown of VMP1 (Figure 6D), an essential gene for zymophagy (Grasso et al., 2011), suggesting that vesicophagy is independent of VMP1. Thus, we concluded that vesicophagy is a previously uncharacterized form of selective autophagy pathway, different from crinophagy or zymophagy.

### Short-Term Autophagy Inhibition Rescues Insulin Storage and Glucose Intolerance in Becn1^F121A^ Mice

**Figure 7:**
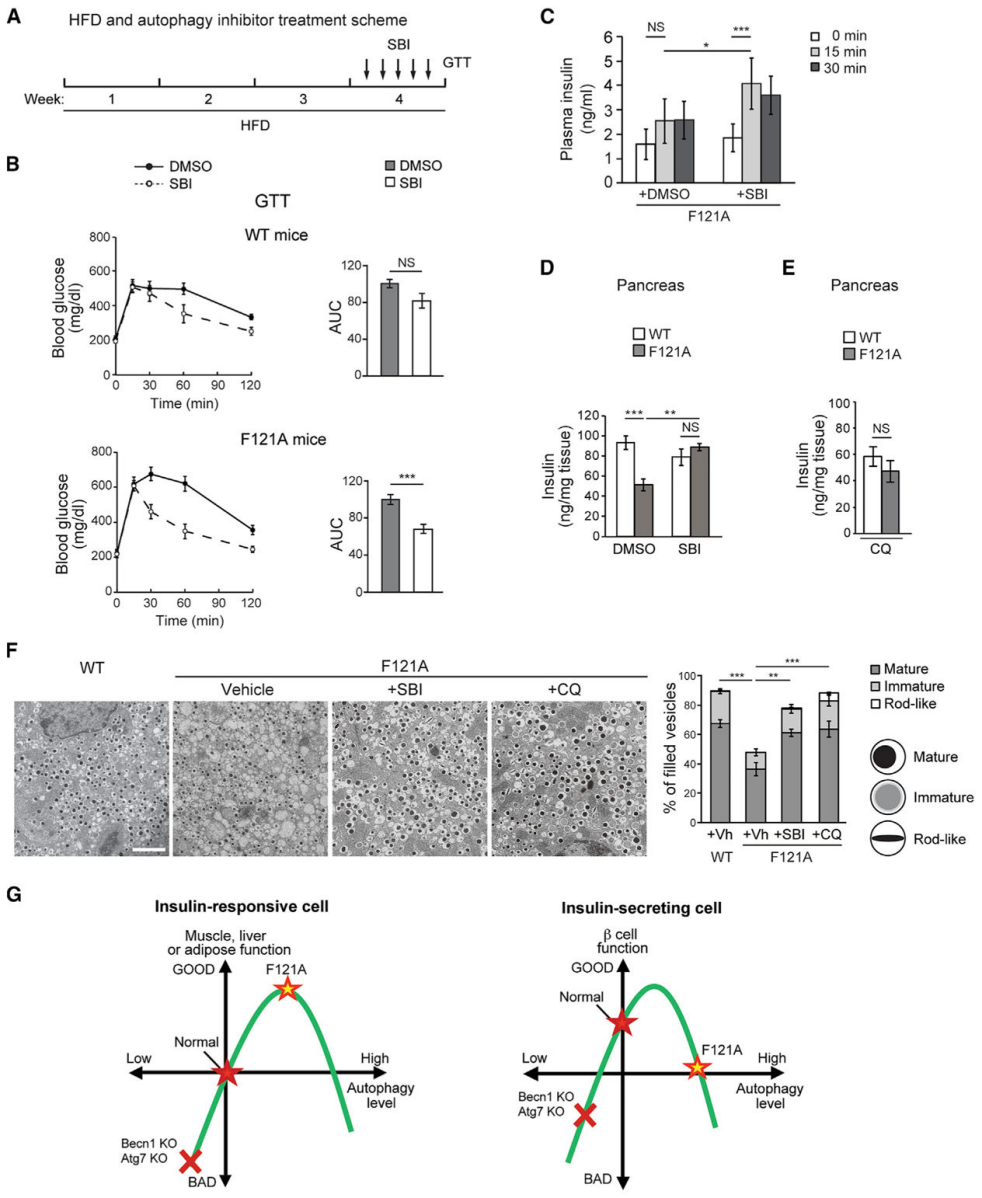
Transient Inhibition of Autophagy Rescues Glucose Intolerance, Insulin Secretion, and Insulin Storage in HFD-fed Becn1^F121A^ Mice (A) Experimental scheme of the rescue experiment. Briefly, mice were treated with HFD for 4 weeks and subject to 5 injections of the autophagy inhibitor SBI-0206965 (SBI) or vehicle (DMSO) once daily in the final week, prior to GTT. (B) Short-term autophagy inhibitor treatment improves glucose tolerance in autophagy-hyperactive mice. GTT of WT (upper panels) or Becn1^F121A^ (lower panels) mice treated with HFD and SBI-0206965 (SBI) or vehicle (DMSO) as in (A). AUC, area under the curve. n = 6–12; t test. ***p < 0.001; t test. (C) ELISA analyses of plasma insulin levels at 0 min, 15 min, and 30 min after glucose injection in WT or Becn1^F121A^ mice treated with HFD and SBI-0206965 (SBI) or vehicle (DMSO) once per day for 5 days. n = 10–13; one-way ANOVA. (D) ELISA analyses of insulin levels in pancreas of WT or Becn1^F121A^ mice treated with HFD and vehicle (DMSO) or SBI-0206965 (SBI) once per day for 5 days. n = 5–9; one-way ANOVA. (E) ELISA analyses of insulin levels in pancreas of WT or Becn1^F121A^ mice treated with HFD and chloroquine (CQ) once per day for 5 days. n = 5–8; one-way ANOVA. (F) Representative EM images (left) and quantification (right) of insulin granules in islets of HFD-fed WT or Becn1^F121A^ mice treated with vehicle, SBI-0206965 (SBI), or chloroquine (CQ) once per day for 5 days. The number of mature, immature, and rod-like insulin granule vesicles was categorized. Scale bar, 2 μm. n = 3; one-way ANOVA. Data represent mean ± SEM. *p < 0.05; **p < 0.01; ***p < 0.001; NS, not significant. (G) Two different rheostat models of autophagy in insulin-producing β cells versus insulin-responsive tissues (such as muscle, liver, and adipose tissue). In insulin-responsive metabolic organs, relatively higher autophagy caused by Becn1^F121A^ gain of function (yellow star) is beneficial for insulin sensitivity, yet excessive autophagy may cause cell death or malfunction. In comparison, in β cells the curve is shifted to the left due to their unique insulin-secretory function, which is also regulated by autophagy. In either tissue, autophagy deficiency caused by deletion of autophagy genes (*Becn1* or *Atg7*, red cross) impairs the physiological function.

To further test the hypothesis that the decline of insulin secretion in HFD-fed Becn1^F121A^ mice is attributed to hyperactive autophagic degradation of insulin granules, we performed rescue experiments using short-term treatment of autophagy inhibitors that block the early steps of autophagosome formation. Briefly, we treated WT and Becn1^F121A^ mice with HFD for 4 weeks; during the last week of HFD treatment, we injected the mice with SBI-0206965, an inhibitor of the autophagy-required ULK1 kinase (Egan et al., 2015), once daily for a total of 5 days (Figure 7A). We found that this transient inhibition of autophagy in Becn1^F121A^ mice by SBI-0206965 significantly rescues glucose tolerance in GTT, whereas, in WT mice, the effect of SBI-0206965 is minimal (Figure 7B). Consistently, we found that treatment of the autophagy inhibitor SBI-0206965 rescued the level of GSIS in the serum 15 min after glucose injection in Becn1^F121A^ mice (Figure 7C) and restored the insulin contents in the pancreas of Becn1^F121A^ mice by ELISA and EM analyses (Figures 7D and 7F). Consistently, chloroquine, a lysosomal inhibitor, also rescued the level of insulin granules in islets of HFD-fed Becn1^F121A^ mice to the WT level, analyzed by ELISA (Figure 7E) and EM (Figure 7F); although, as previously reported (Andersson et al., 1980; Chatterjee and Schatz, 1988), we found that chloroquine treatment impairs insulin granule maturation, resulting in relatively more rod-like crystals of insulin (Figure 7F). Thus, altogether, these data suggest that transiently inhibiting autophagy in Becn1^F121A^ mice restores insulin secretion by increasing the insulin storage in pancreas.

Taken together, we propose a model in which insulin-responsive cells and insulin-producing β cells require different levels of autophagy for their optimal function (Figure 7G): in insulin-responsive tissues, relatively higher autophagy mediated by Becn1^F121A^ is beneficial for insulin sensitivity, whereas in β cells, due to their insulin-secretory function, too much autophagy impairs insulin secretion by degrading insulin granules and reducing insulin storage. Thus, our findings implicate that we should transiently or periodically activate autophagy to increase insulin sensitivity in insulin-responsive tissues without affecting the insulin load in islets (or increase both insulin sensitivity and β cell function), in future therapeutic development against T2D via modulating the autophagy activity.

## DISCUSSION

Utilizing mouse models with different autophagy levels is necessary to understand the function of autophagy. To achieve this goal, we generated a genetic model of autophagy hyperactivation, the Becn1^F121A^ knockin mouse model. We found that these mice show high basal autophagy in β cells, to a level comparable to that of fasting-induced autophagy. Notably, suppression of autophagy has been reported in β cells after 4 hr of starvation (Goginashvili et al., 2015); yet it is a much shorter period of starvation compared to the starvation time for autophagy detection *in vivo* in our studies and others’ (24–48 hr) (Fujimoto et al., 2009) (Figure 1C). Thus, we suspect that autophagy suppression in islets after 4-hr fasting may be a systematic metabolic response to acute (4-hr) starvation. Furthermore, we deduced that, functionally, starvation-induced autophagy in β cells may play an important role in maintaining the proteostatic balance and degrading unnecessary insulin granules, when the blood glucose is low and insulin secretion is not required under starvation conditions.

Interestingly, in response to an HFD challenge, these autophagy-hyperactive mice are more glucose intolerant, but are more insulin sensitive, than mice with normal levels of autophagy. We further discovered that Becn1^F121A^-induced autophagy reduces impaired insulin secretion by reducing insulin storage in the islets. Importantly, we demonstrated that, under normal conditions, insulin granules are not efficient autophagic cargos, whereas upon autophagy activation, insulin granules are sequestered by autophagosomes and degraded by Becn1-mediated chronic autophagy in β cells, which can be rescued by short-term treatment of autophagy inhibitors.

This autophagic degradation of insulin granules we characterized here is mechanistically different from “crinophagy.” The latter was described more than 30 years ago (Orci et al., 1984) and refers to the disposal of nascent hormone-containing secretory vesicles by direct fusion with lysosomes (Figure 6E). Whether autophagy genes are involved in crinophagy is not clear. Here, we propose a broader concept of “vesicophagy,” which is the autophagic degradation of secretory vesicles that contain various granular contents (Figure 6E). Notably, in vesicophagy, we define that not only endocrine or exocrine hormones but also other secreted contents, such as neurotransmitters, may be degraded through the autophagy-dependent pathway. This concept is also indirectly supported by previous reports that transient inhibition of mammalian target of rapamycin (mTOR), a major upstream inhibitor of autophagy, by rapamycin reduced GSIS from rat islets (Fraenkel et al., 2008); whereas depletion of TSC2, an upstream suppressor of mTOR, leads to mTOR upregulation and increases insulin secretion in *Tsc2* KO mice and *Tsc2* knockdown Ins-1 β cells (Koyanagi et al., 2011). However, direct evidence was lacking on the degradative role of autophagy in the regulation of insulin homeostasis in β cells or whole animals. Thus, here, we presented important experimental evidence that chronic autophagy hyperactivation impairs glucose tolerance by sequestrating and degrading insulin granules upon HFD challenge. It should be noted that exacerbated glucose intolerance is observed only in HFD-fed, but not normal diet-fed, Becn1^F121A^ mice, suggesting that the impairment in insulin storage is more apparent only when the animals become pre-diabetic and the peripheral demand for insulin is increased. In addition, different from type 1 diabetes, high autophagy does not cause islet destruction; instead, β cell mass expands in autophagy-hyperactive mice, possibly due to a compensatory effect of reduction in insulin contents.

On the other hand, we found that, despite decreased insulin storage, autophagy-hyperactive Becn1^F121A^ mice showed improved insulin sensitivity compared to WT mice in response to HFD. We demonstrated that reduced ER stress observed in Becn1^F121A^ mice is an underlying mechanism by which autophagy hyperactivation increases insulin sensitivity in insulin-responsive tissues (Figure 3). Consistent with this hypothesis, autophagy deficiency caused by knockdown of Atg7 in liver has been associated with elevated hepatic ER stress and insulin resistance (Yang et al., 2010). It is possible that other metabolic changes caused by the Becn1^F121A^ mutation, such as enhanced mitophagy (Figure S4B), may also contribute to insulin sensitization, which requires further investigation.

Thus, we proposed that autophagy regulates the function of β cells and insulin-responsive tissues differently and developed a rheostat model in which the metabolism-autophagy curve is shifted toward left in β cells compared to insulin-responsive metabolic organs (Figure 7G). In this model, autophagy has biphasic effects in diabetes, depending on the cell types: activation of autophagy in insulin-responsive tissues such as muscle, liver, and adipose tissues improves insulin response and function; whereas in β cells, chronic autophagy activation reduces insulin secretion caused by excessive degradation of insulin granules. Based on this model, we propose that autophagy inducers should be applied acutely or periodically to improve both insulin sensitivity and β cell function or increase insulin sensitivity in insulin-responsive tissues without decreasing insulin storage in β cells. Indeed, supporting this hypothesis, we found that treatment of an autophagy inducer, Rg2, for no more than 4 weeks improves both insulin sensitivity and glucose tolerance in HFD-fed WT mice (Fan et al., 2017), suggesting that a short term (4 weeks) of autophagy induction is more metabolically beneficial than long-term or lifelong autophagy induction (e.g., 16 weeks, as used in this study in Becn1^F121A^ mice). Thus, for potential future therapeutic interventions based on autophagy modulation, precise or tissue-specific regulation of autophagy activity is necessary to optimize insulin sensitivity, glucose tolerance, and β cell function. In addition, generating tissue-specific autophagy-hyperactive mouse models will be useful to better understand the function of autophagy in different metabolic tissues during the pathogenesis of T2D.

## EXPERIMENTAL PROCEDURES

Further details on the procedures and resources can be found in the Supplemental Experimental Procedures.

### Mice

All mice used in the study were male adult mice in the C57BL/6J background. GFP-LC3, Becn1^+/−^ KO, and Becn1^F121A/F121A^ mice were described previously (Mizushima et al., 2004; Qu et al., 2003; Rocchi et al., 2017). Becn1^F121A/F121A^ mice were backcrossed for more than 12 generations to C57BL/6J mice. Becn1^+/+^, Becn1^F121A/+^, and Becn1^F121A/F121A^ mice used in the study were littermates from Becn1^F121A/+^ intercrosses. db/db mice were purchased from the Jackson Laboratory (#000697, B6.BKS(D)-*Lepr^db^*/J) in a C57BL/6J background. All mice were housed on a 14-hr/10-hr light/dark cycle. All animal protocols were approved by the Northwestern University Institutional Animal Care and Use Committee (IACUC). The HFD (A12492) was purchased from Research Diets (New Brunswick, NJ, USA). Insulin (2 U/kg body weight) and SBI-0206965 (2 mg/kg body weight) were injected into mice intraperitoneally (i.p.).

### GTT and ITT

GTT and ITT procedures were modified from previously reported protocols (Fan et al., 2017; He et al., 2012a). Mice were fasted for 6 hr and 4 hr prior to GTT and ITT, respectively. Glucose was i.p. injected at 1.5 g/kg body weight in HFD-fed mice and 0.75 g/kg in db/db mice. Mouse insulin was i.p. injected at 0.75 U/kg body weight. Blood was collected from the tail vein, and glucose concentration was determined using alphaTRAK 2 (Zoetis, Parsippany, NJ, USA). The insulinogenic index was calculated as follows: insulinogenic index = (blood insulin at 15 min – blood insulin at 0 min [picomoles per liter])/(blood glucose at 15 min – blood glucose at 0 min [milligrams per deciliter]).

### Islet Isolation

Islets were isolated from 4 WT or Becn1^F121A^ knockin mice expressing GFP-LC3. After dissection, Clzyme-RI (Vitacyte, Indianapolis, IN, USA) was injected into the pancreas through the bile duct ampullae, and pancreas samples were collected and digested for 17 min at 37°C with shaking every 5 min (Kulkarni et al., 1997). Subsequently, ice-cold Hank’s balanced salt solution (HBSS) (Thermo Fisher Scientific, #24020117) was added, and samples were centrifuged at 150 × *g* for 2 min at 4°C. Pellets were resuspended with HBSS, filtered through a 400-μm filter mesh, and centrifuged at 150 × *g* for 2 min at 4°C. Pellets were then resuspended in Histopaque 1119 (Sigma-Aldrich), and Histopaque 1077 (Sigma-Aldrich) and HBSS were sequentially added to make a 3-layer solution in a falcon tube. After centrifugation at 1,400 × g for 30 min at room temperature, monolayered islet cells were collected from the middle layer containing Histopaque 1077 and washed twice with ice-cold HBSS.

### Immunoprecipitation of Autophagosomes

Purified islets or liver samples from GFP-LC3 mice were resuspended in lysis buffer (0.25 M sucrose, 1 mM EDTA, and 10 mM HEPES-NaOH [pH 7.4]) supplemented with protease inhibitor and phosphatase inhibitor cocktail (Thermo Fisher Scientific) and homogenized by a Dounce homogenizer. Cell debris and nucleus were removed by centrifugation at 1,000 × *g* for 10 min at 4°C. The supernatant was then centrifuged at 20,000 × *g* for 20 min at 4°C to enrich autophagosomes. Pellets containing autophagosomes were resuspended in lysis buffer and incubated with anti-GFP-antibody or normal rabbit immunoglobulin G (IgG)-conjugated Dynabeads (Life Technologies, 10004D) for 2 hr at 4°C. The beads were washed four times with lysis buffer supplemented with 0.15 M NaCl and boiled in SDS sample buffer for western blot analysis.

### Insulin Secretion Assay

For MIN6 β cells, 2 × 10^4^ cells were inoculated in 96-well plates and were incubated for 7 days. Cells were washed once with incubation buffer (0.1% [v/v] BSA-supplemented Krebs-Ringer HEPES buffer; 130 mM NaCl, 4.7 mM KCl, 0.5 mM NaH_2_PO_4_, 0.5 mM MgCl_2_, 1.5 mM CaCl_2_, 10 mM HEPES-NaOH [pH 7.4]) and incubated in the incubation buffer supplemented with 2.8 mM or 16.7 mM glucose. After 2 hr, cells were incubated in fresh incubation buffer containing 2.8 mM or 16.7 mM glucose for another 2 hr and then in fresh incubation buffer containing 2.8 mM or 16.7 mM glucose for 1 hr. After incubation, supernatant containing secreted insulin was collected by centrifugation at 1,500 × *g* at 4°C for 5 min. The level of insulin was subsequently measured by ELISA (EZRMI-13k; EMD Millipore, Billerica, MA, USA). For isolated islets, after isolation, hand-picked islets were recovered in RPMI 1640 media (Thermo Fisher Scientific) supplemented with 10% FBS, 100 U/mL penicillin, and 100 μg/mL streptomycin for up to 72 hr. For GSIS experiments, islets after 24-hr recovery were used. Islets were incubated with incubation buffer (0.1% [v/v] BSA-supplemented Krebs-Ringer buffer-HEPES; 130 mM NaCl, 4.7 mM KCl, 0.5 mM NaH_2_PO_4_, 0.5 mM MgCl_2_, 1.5 mM CaCl_2_, 10 mM HEPES-NaOH [pH 7.4]) for 30 min at 37°C and then incubated with incubation buffer supplemented with 2.8 mM glucose for 1 hr at 37°C. Five islets comparable in size were transferred to 1 mL incubation buffer supplemented with 2.8 mM or 16.7 mM glucose in glass tubes and incubated for 1 hr at 37°C with shaking every 5 min. After incubation, samples were cooled quickly on ice for 15 min, and five islets and supernatant were collected. For intra-islet insulin extraction, the five islets were dissolved in 500 μL of 180 mM HCl containing 0.1% Triton X-100 and 70% ethanol, sonicated for 10 s, and then incubated overnight (O/N) at 4°C. The level of insulin was subsequently measured by ELISA. Insulin secretion was calculated as follows: percent secreted insulin = (insulin in supernatant [in nanograms]/[insulin in supernatant + intra-islet insulin, in nanograms]) × 100.

### Analysis of Insulin Secretion by Hyperglycemic Clamp

Hyperglycemic clamp studies were performed at the National Mouse Metabolic Phenotyping Center (MMPC) at University of Massachusetts Medical School as previously described (Fergusson et al., 2014; Perry et al., 2016). Briefly, WT and Becn1^F121A^ mice fed with 9-week HFD were fasted overnight, and a 2-hr hyperglycemic clamp was conducted in the conscious mice with a variable infusion of 20% glucose to increase and maintain plasma glucose concentrations at ~350 mg/dL. Blood samples at 10 min, 15 min, and 20 min intervals were collected to measure plasma glucose and insulin concentrations.

### Insulin Measurement in Pancreas

Pancreas samples were collected and homogenized in acid-ethanol solution (0.2 M HCl in ethanol) at 4°C overnight. Supernatant was collected by centrifugation at 4,000 rpm for 20 min at 4°C. The level of insulin was measured by ELISA (#90082; Crystal Chem, Elk Grove Village, IL, USA).

### Transmission EM

Pancreas and liver samples were collected and fixed with fix solution (2% v/v paraformaldehyde, 2.5% v/v glutaraldehyde, and 0.1 M sodium cacodylate [pH 7.3]) at room temperature overnight. EM analysis was carried out using a Tecnai Spirit G2 electron microscope (FEI; Hillsboro, OR, USA).

### Statistical Analysis

p ≤ 0.05 was considered statistically significant in a one-way ANOVA or two-tailed, unpaired Student’s t test for detection of differences between two experimental groups. Data in the figures are depicted as mean ± SEM.

## Supplementary Material



## Highlights

- Becn1^F121A^ hyperactivates autophagy in multiple metabolic tissues
- Becn1-activated autophagy improves insulin sensitivity but impairs glucose tolerance
- High autophagy sequesters insulin granules and reduces insulin storage in β cells
- Transient autophagy inhibition rescues glucose intolerance in Becn1^F121A^ knockin mice
