# Supplementary material for: Autophagy Differentially Regulates Insulin Production and Insulin Sensitivity
Source: Cell Rep. Author manuscript; Available in PMC 2018 Jul 22. (PMC6054876; doi:10.1016/j.celrep.2018.05.032)
Supplement: Supplementary file 1 [file NIHMS976520-supplement-supplement_1.pdf]

Cell Reports, Volume *23*

Supplemental Information

Autophagy Differentially Regulates

Insulin Production and Insulin Sensitivity

Soh Yamamoto, Kenta Kuramoto, Nan Wang, Xiaolei Situ, Medha Priyadarshini, Weiran Zhang, Jose Cordoba-Chacon, Brian T. Layden, and Congcong He

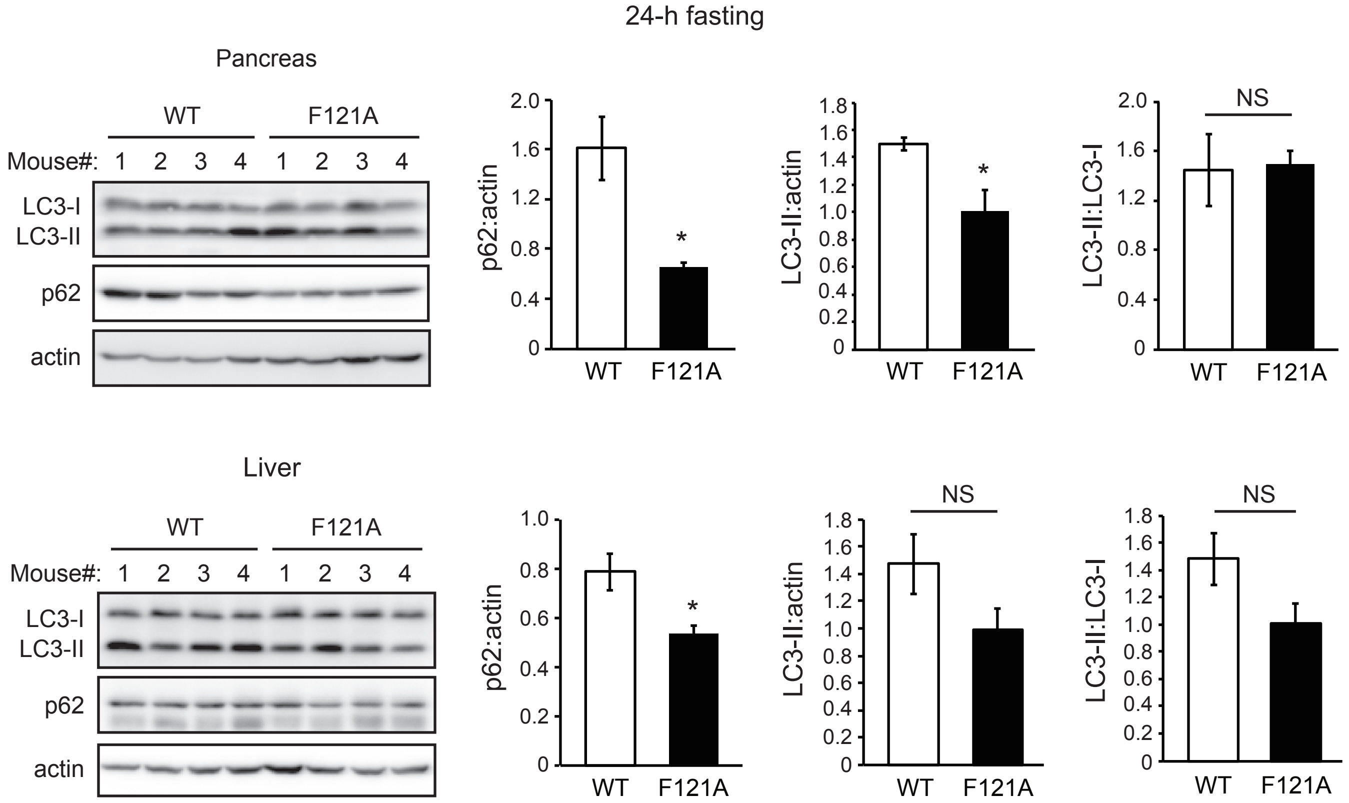

Figure S1. Autophagy markers under the starvation condition in Becn1^{F121A} mice compared to WT mice. Related to Figure 1.

Western blot analysis (left) and quantification (right) of autophagy markers p62, LC3-I and LC3-II in pancreas (upper) and liver (lower) of WT and Becn1^{F121A} mice after 24-h fasting. One-way ANOVA. N=4. *, $P<0.05$; NS, not significant.

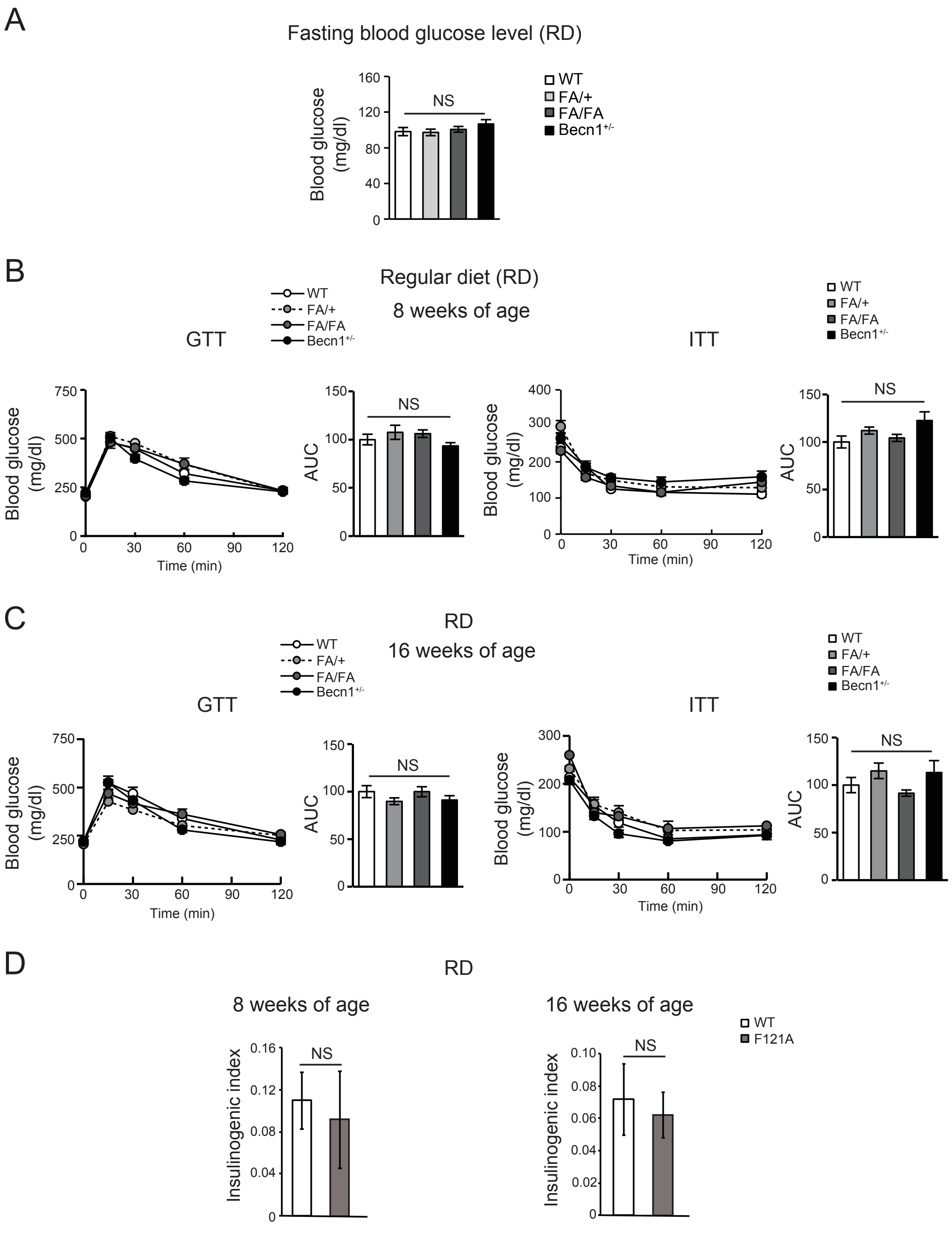

Figure S2. Autophagy-hyperactive Becn1^{F121A} mice show normal fasting blood glucose, glucose tolerance, insulin tolerance and insulinogenic index under regular diet feeding. Related to Figure 2.

Fasting blood glucose levels (A), GTT and ITT (B-C), and insulinogenic index (D) of 8-week old and 16-week old Becn1$^{+/+}$ (WT), Becn1$^{FA/+}$ (FA/+), Becn1$^{FA/FA}$ (FA/FA), or Becn1$^{+/-}$ KO mice fed with regular diet. Data represent mean ± s.e.m. One-way ANOVA with Dunnett's test. (A) N=5-10; (B) N=8-14; (C) N=7-8; (D) N=10-11. NS, not significant. FA, F121A.

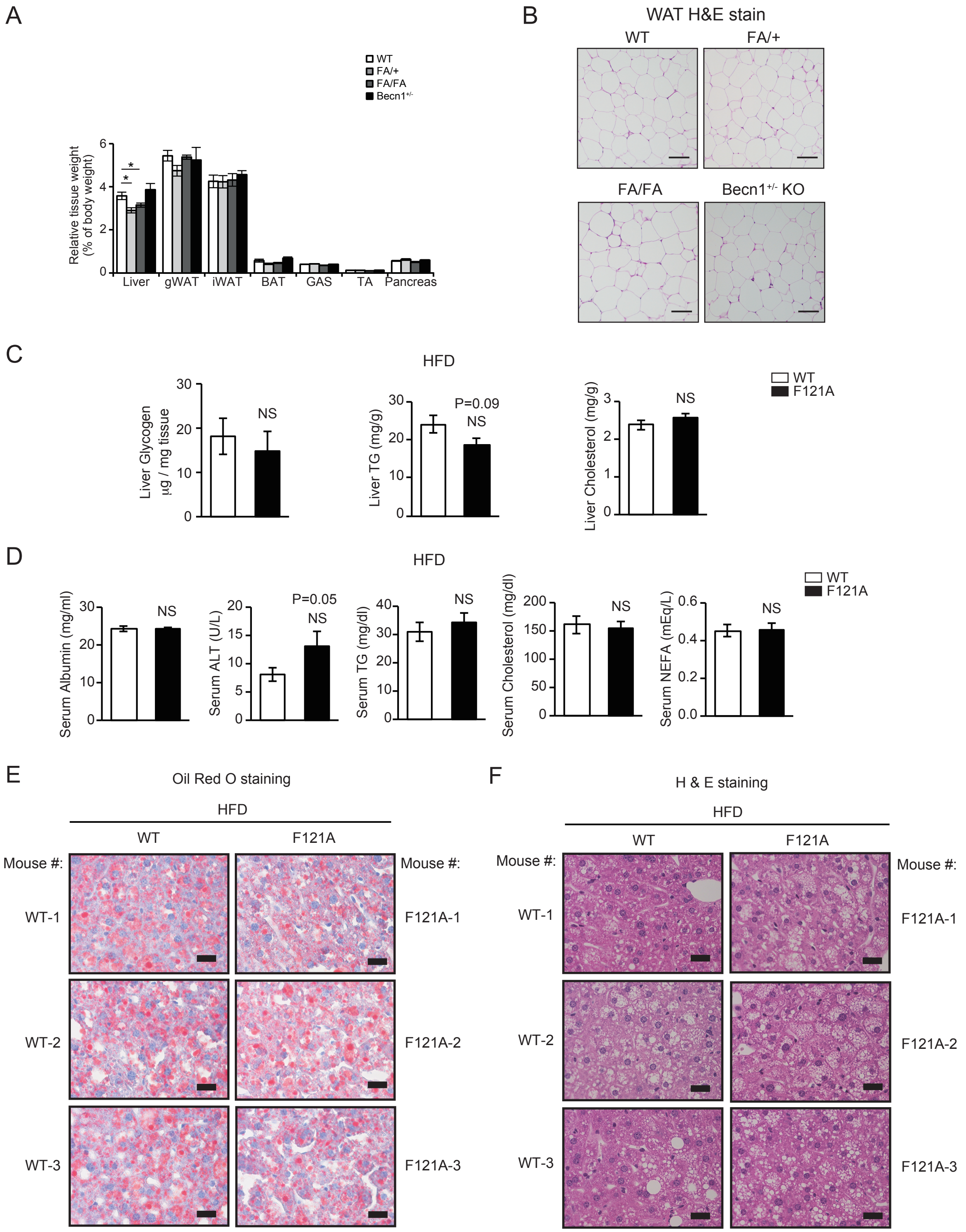

Figure S3. Except a slight reduction in liver weight, no significant alterations in weight of major metabolic organs, hepatic glycogen levels, lipid contents and morphology, and serum lipid, albumin and ALT levels, were observed in HFD-fed Becn1^{F121A} mice, compared to HFD-fed WT mice. Related to Figure 2.

(A) Weight of indicated organs in the above mice after 8-week HFD feeding. gWAT, gonadal white adipose tissue; iWAT, inguinal white adipose tissue; BAT, brown adipose tissue. GAS, gastrocnemius; TA, tibialis anterior. N=8-13. One-way ANOVA. (B) H&E stain reveals no change in white adipocyte size or morphology in autophagy-hyperactive or -deficient mice. (C) Levels of TGs (triglycerides), cholesterol and glycogen in liver of 8-week HFD-fed WT and Becn1^{F121A} mice. (D) Levels of TGs (triglycerides), cholesterol, NEFAs (Non-esterified fatty acids), albumin and ALT (alanine transaminase) in serum of 8-week HFD-fed WT and Becn1^{F121A} mice. (E-F) Oil Red O staining (E) and H&E staining (F) of liver from 8-week HFD-fed WT and Becn1^{F121A} mice. Representative images from 3 mice of each genotype are shown. Scale bar, 100 μm. Data represent mean ± s.e.m. One-way ANOVA with Dunnett's test. *, P<0.05; NS, not significant.

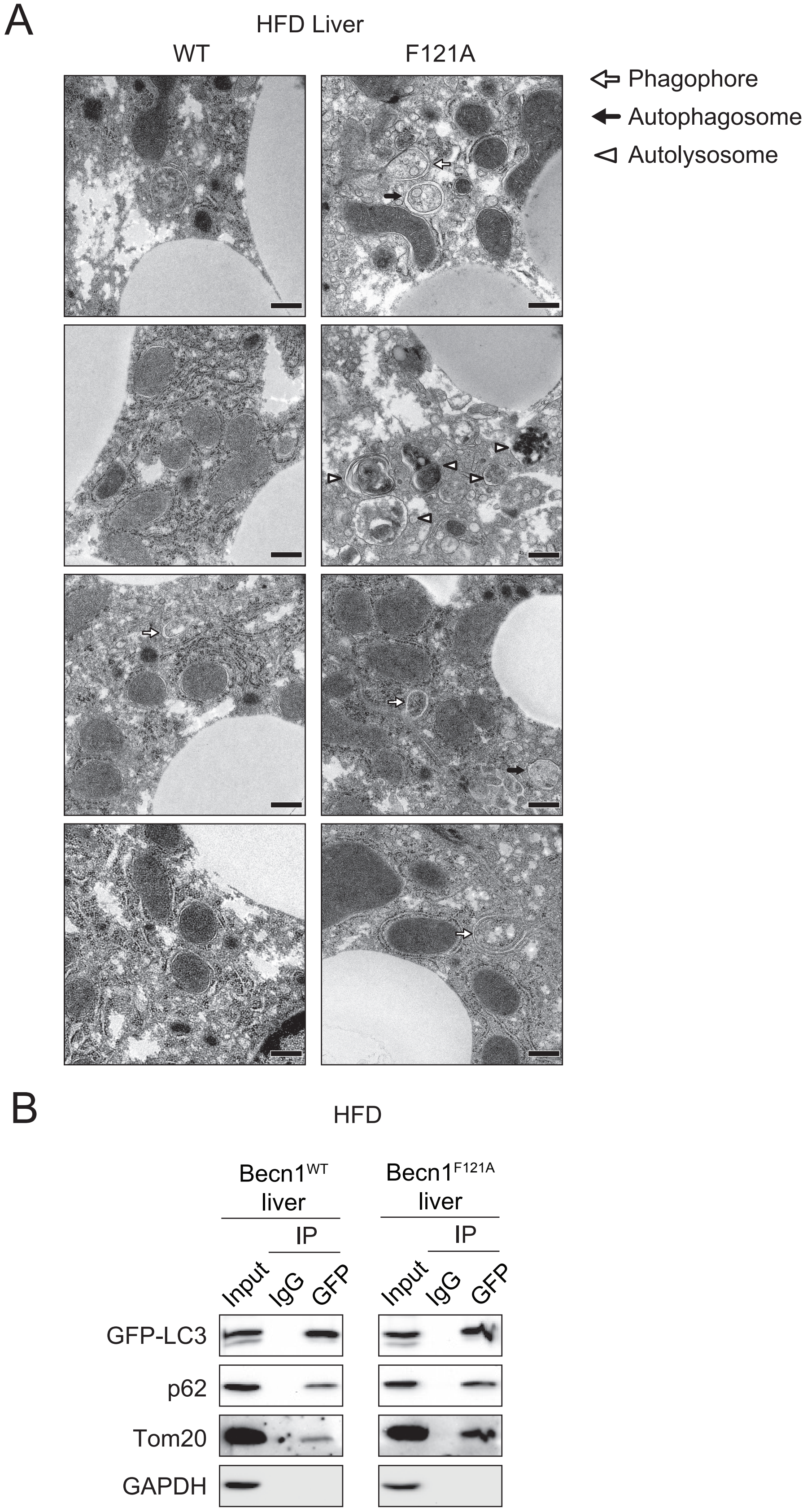

Figure S4. Enhanced bulk autophagy and mitophagy in the liver of HFD-fed Becn1^{F121A} mice, compared to HFD-fed WT mice. Related to Figures 1 and 2.

(A) EM analyses of autophagosomal structures (phagophores, autophagosomes and autolysosomes) in the liver of 8-week HFD-fed male WT and Becn1^{F121A} mice. Representative images from 3 different mice of each genotype were shown. Scale bar, 500 nm. (B) Western blot detection of the mitochondrial membrane protein Tom20 in immunoisolated autophagosomes by GFP antibody, from liver lysates of GFP-LC3 mice expressing WT Becn1 or Becn1^{F121A} fed with HFD for 8 weeks. GFP and p62 serve as positive controls, and GAPDH serves as a negative control.

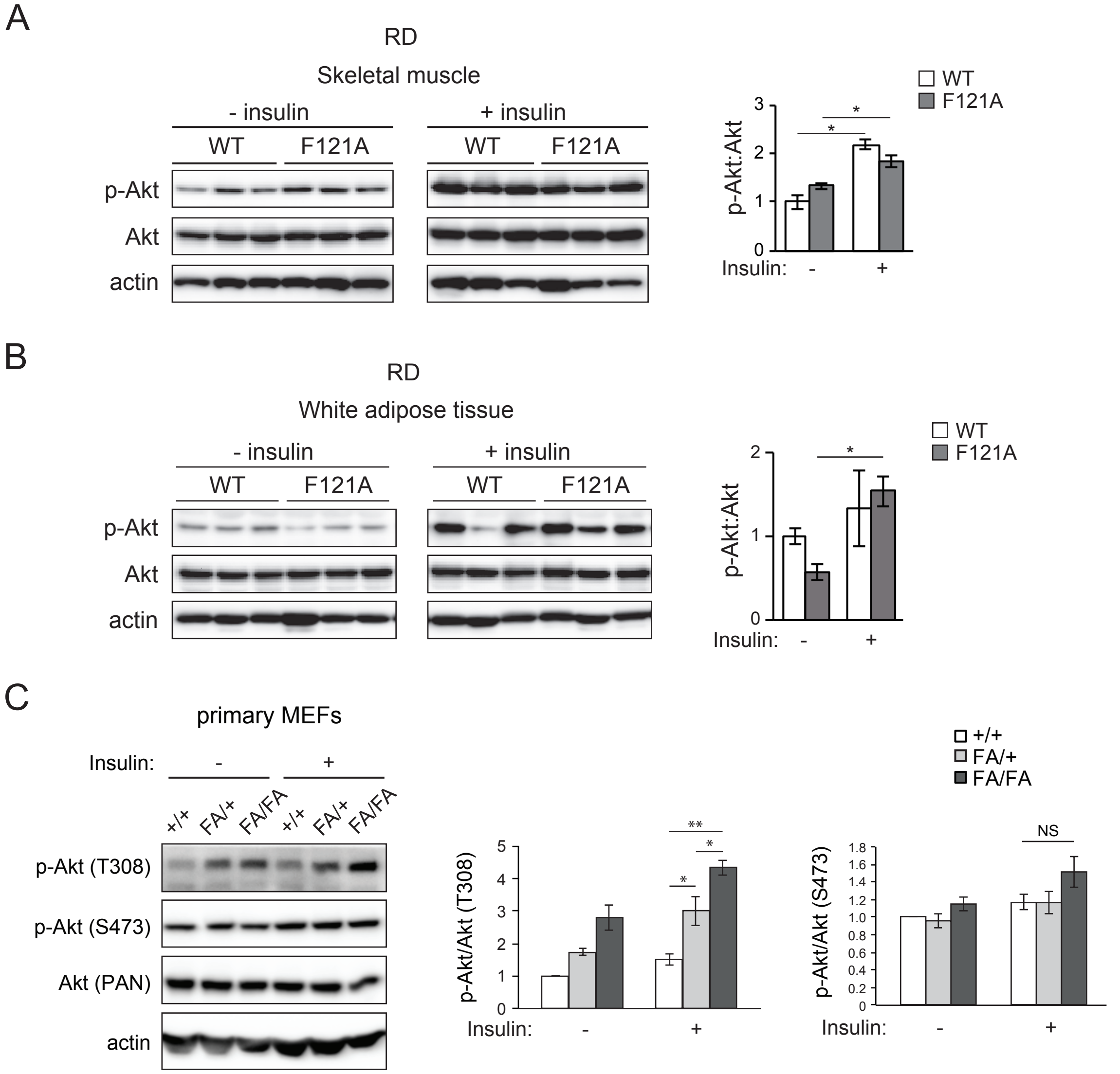

Figure S5. Insulin signaling in regular diet (RD)-fed Becn1^{F121A} mice and in Becn1^{F121A} primary MEFs. Related to Figure 3.

(A-B) Western blot analyses (left) and quantification (right) of insulin-induced Akt T308 phosphorylation in muscle (A) and white adipose tissue (B) of 16-week old WT and Becn1^{F121A} mice fed with RD. The tissues were collected 10 min after i.p. injection of 2 U/kg insulin. N=3. One-way ANOVA with Tukey-Kramer test. (C) Western blot analysis (upper) and quantification (lower) of insulin-stimulated phosphorylation of Akt T308 and S473 after 5-min insulin treatment in primary MEFs of Becn1$^{+/+}$ (WT), Becn1$^{FA/+}$ (FA/+), or Becn1$^{FA/FA}$ (FA/FA) mice. Results represent mean ± s.e.m. N=4. T test.*, P<0.05; **, P<0.01; NS, not significant.

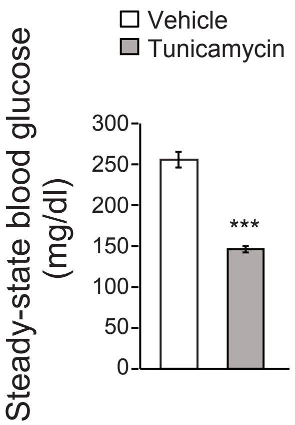

Figure S6. Tunicamycin treatment interferes with gluconeogenesis. Related to Figure 3.
Steady-state blood glucose levels of Becn1^{F121A} mice subject to one injection of vehicle or the ER stressor tunicamycin at 1 μg/kg for 24 h. N=5. t-test. Results represent mean ± s.e.m. ***, P<0.001.

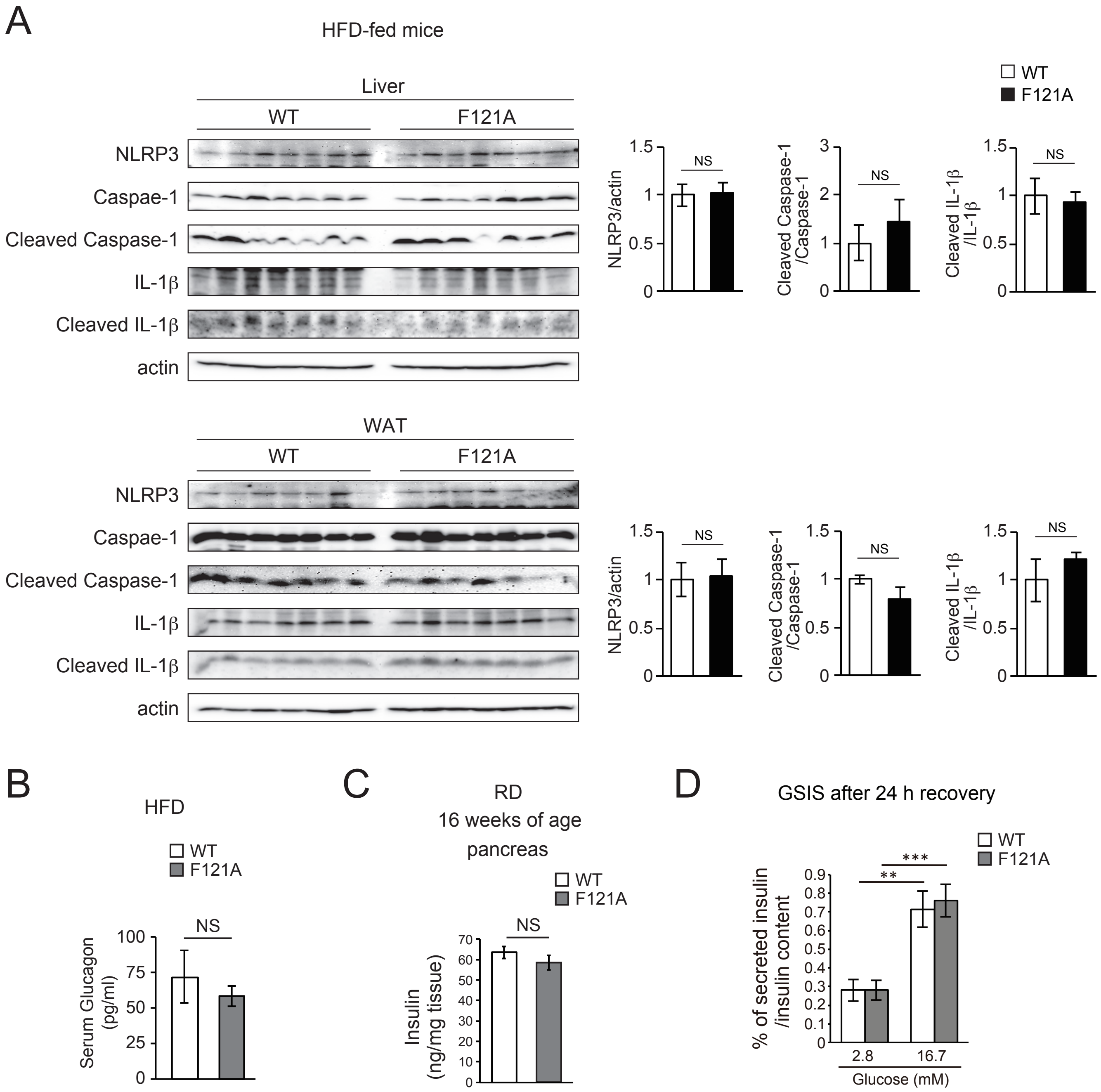

Figure S7. Becn1^{F121A} mice show no alterations in inflammasome activation, glucagon release from α cells, or secretory function of β cells in response to HFD, and no alteration in insulin storage under regular diet (RD) feeding. Related to Figures 3-5.
(A) Western blot analysis and quantification of inflammasome markers NLRP3, full-length and cleaved Caspase-1, and full-length and cleaved IL-1β in liver and white adipose tissue (WAT) of WT and Becn1^{F121A} (F121A) mice fed with HFD for 8 weeks. N=7. (B) ELISA analysis of serum glucagon levels in 8-week HFD-fed WT and Becn1^{F121A} mice. N=9. (C) ELISA analysis of insulin contents in pancreas of 16-week old WT and Becn1^{F121A} mice fed with RD. N=7. (D) Percentage of secreted insulin versus insulin storage in islets isolated from HFD-fed WT and Becn1^{F121A} mice. GSIS from isolated islets was performed after 24-h ex vivo culture in RPMI1640 media after isolation. N=9-10, t-test. **, $P<0.01$; ***, $P<0.001$; NS, not significant.

Supplemental Experimental Procedures

Antibodies and reagents

The following antibodies were used in Western blot analyses: anti-LC3 (NB100-2220; Novus Biologicals), anti-p62 (610833; BD Biosciences), anti-GFP (G1544; Sigma-Aldrich, St. Louis, MO, USA), anti-GAPDH (MA5-15738; Thermo Fisher Scientific), anti-Caspase-1 (5B10; eBioscience), anti-Becn1 (H300) (sc-11427), HRP-conjugated anti-β actin (sc-47778), anti-Tom20 (FL145), and anti-S16 ribosomal protein (T-19) antibodies (Santa Cruz Biotechnology), anti-Lamp1 (D2D11), anti-phospho-AKT (T308) (C67E), anti-phospho-AKT (S473) (D9E), anti-pan-AKT (D25E6), anti-NLRP3 (D4D8T), anti-IL-1β (D6D6T), anti-cleaved IL-1β (Asp117), anti-Bip (C50B12), anti-C/EBP homologous protein (CHOP) (L63F7), anti-HA (C29F4), anti-VMP1 (D1Y3E), anti-COX IV (4D11-B3-E8), and S6 Ribosomal protein (5G10) antibodies (Cell Signaling Technology), and anti-glucagon (ab36232), anti-insulin (ab181547), and FAM134B (ab151755) antibodies (Abcam). SBI-0206965 and tunicamycin were purchased from Cayman Chemical and R&D Systems, respectively, and dissolved in DMSO. Recombinant human insulin was purchased from Thermo Fisher Scientific (12585-014).

Cell culture

MIN6 cells were obtained from AddexBio, Human embryonic kidney (HEK) 293 FT cells were obtained from ATCC. MIN6 were maintained in Dulbecco's modified eagle medium (DMEM) supplemented with 10% (v/v) fetal bovine serum (FBS), 100 U/ml penicillin, 100 μg/ml streptomycin, 2 mM L-glutamine, 2 mM sodium pyruvate, and 0.001% (v/v) β-mercapto ethanol. HEK293 FT cells were maintained in DMEM supplemented with 10%(v/v) FBS, 100 U/ml penicillin and 100 μg/ml streptomycin. Primary mouse embryonic fibroblasts (MEFs) were maintained in DMED supplemented with 20% (v/v) FBS, 100 U/ml penicillin, 100 μg/ml streptomycin, 2 mM L-glutamine, 1x non-essential amino acid solution (Thermo Fisher Scientific), and 0.001% (v/v) β-mercapto ethanol. For insulin sensitivity experiments, cells were incubated in DMEM containing 0.5% (v/v) FBS overnight, prior to stimulation with 200 nM human insulin for 5 min or 10 min.

siRNA transfection

Silencer select siRNAs (*Atg5*: s62452; *Atg7*: s92536; *Vmp1*: s93986) were purchased from Thermo Fisher Scientific. siRNA was transfected using Lipofectamine 2000 (Thermo Fisher Scientific) following the reverse transfection protocol. 0.5×106 cells were cultured with 20 pmol siRNA/Lipofectamine 2000 complex in 12-well plates for 72 h.

Plasmid construction and generation of Becn1^{F121A}-expressing stable cells

Mouse wild-type (WT) Becn1 or Becn1^{F121A} mutant cDNA was sub-cloned into the pCDH-CMV-MCS-EF1-GreenPuro vector (System Biosciences, Palo Alto, CA, USA) using XbaI and BamHI restriction sites. Lentivirus encoding Becn1 or Becn1^{F121A} was produced by co-transfection with packing plasmids, pCMV-VSV-G (addgene, #8454) and psPAX2 (addgene, #12260), in HEK293 FT cells. The generated lentivirus encoding Becn1 or Becn1^{F121A} were used to infect MIN6 cells at the multiplicity of infection of 1 for 24 h in the presence of 8 μg/ml polybrene (Santa Cruz Biotechnology). Infected cells were selected and maintained in DMEM supplemented with 2 μg/ml puromycin (Thermo Fisher Scientific).

Microscopy

Immunofluorescence (IF) staining was performed using frozen tissue sections. Tissue sections were immunostained with anti-insulin (1:1000), anti-glucagon (1:200), or anti-Lamp1 (1:200) antibodies overnight at 4°C. Alexa Fluor 488 anti-sheep IgGs (Thermo Fisher Scientific, A-11015, 1:750) or Alexa Fluor 594 anti-rabbit IgGs (Thermo Fisher Scientific, A11012, 1:750) were used as the secondary antibody. All antibodies were diluted in PBS containing 2% (v/v) bovine serum albumin (BSA) (Thermo Fisher Scientific, BP1605). Immunostained sections were embedded in the VECTASHILD mounting media (Vector Laboratory, Burlingame, CA, USA, H1200), and analyzed by a Nikon ECLIPSE Ti-E Inverted Microscope (Nikon, Melville, New York, USA).

Quantitative real-time PCR (qRT-PCR)

Total RNA was purified using TRIzol reagent (Thermo Fisher Scientific) according to the manufacturer's protocol. cDNA library was synthesized using SuperScript III (Thermo Fisher Scientific). The following primer sets for mouse *Insulin 1* (*Ins1*)-Forward: 5'-CCTTCAGACCTTGGCGTTGG-3'; Reverse: 5'-CGAGGTGGGCCTTAGTTGCA-3'), mouse *Insulin 2* (*Ins2*) (Forward: 5'-ACCCACAAGTGGCACAACTG-3'; Reverse: 5'-AGGGGTAGGCTGGGTAGTG-3'), mouse *Atf6* (Forward: 5'-GGACGAGGTGGTGTCAGAG-3'; Reverse: 5'-GACAGCTCTTCGCTTTGGAC-3'), mouse *Gapdh* (Forward: 5'-AACTTTGGCATTGTGGAAGG-3';

Reverse: 5'-GGATGCAGGGATGATGTTCT-3'), and mouse β-actin (Forward: 5'-GATCTGGCACCACACCTTCT-3'; Reverse: 5'-GGGGTGTTGAAGGTCTCAAA-3') were used. qRT-PCR was performed using Powerup SYBR Green Master Mix (Life Technologies Science) on a LightCycler480 system (Roche, Base, Switzerland). Expression of each mRNA was normalized to β-actin and their relative quantity was calculated by ΔΔCt methods.

Analysis of serum and hepatic metabolites
Serum ALT was measured with a colorimetric assay (Liquid ALT (SGPT), #A7526, Pointe Scientific). Serum albumin was measured with the BCG Albumin Assay kit (#MAK124, Sigma-Aldrich). Hepatic glycogen content was measured as previously described (Haemmerle et al., 2011). Serum NEFA, and serum and hepatic TG and cholesterol, were measured with colorimetric assays (Wako Diagnostics) according to manufacturer's instructions. For hepatic lipids (TG and cholesterol), their levels were measured from isopropanol extracts as previously described (Wolf Greenstein et al., 2017): briefly, small pieces (~50 mg) of liver were homogenized in isopropanol (10 mg tissue/100 μl isopropanol), and the supernatants were collected and diluted for the measurement.

Supplemental References

Haemmerle, G., Moustafa, T., Woelkart, G., Buttner, S., Schmidt, A., van de Weijer, T., Hesselink, M., Jaeger, D., Kienesberger, P.C., Zierler, K., et al. (2011). ATGL-mediated fat catabolism regulates cardiac mitochondrial function via PPAR-alpha and PGC-1. Nat Med *17*, 1076-1085.
Wolf Greenstein, A., Majumdar, N., Yang, P., Subbaiah, P.V., Kineman, R.D., and Cordoba-Chacon, J. (2017). Hepatocyte-specific, PPARgamma-regulated mechanisms to promote steatosis in adult mice. J Endocrinol *232*, 107-121.
